# Pellino1-mTOR/S6K1 signaling axis is a key pathogenesis for the development of polycystic kidney disease

**DOI:** 10.1038/s41419-026-08479-6

**Published:** 2026-03-05

**Authors:** Suhyeon Kim, Min-Hee Kim, Bo-Kyoung Ko, Kyung-Mo Kim, Naiyu Wang, Su-Mi Jo, Heounjeong Go, Eun-Ji Park, Chang-Woo Lee

**Affiliations:** 1 Department of Molecular Cell Biology, Sungkyunkwan University School of Medicine, Suwon, Republic of Korea; 2 Department of Pathology, Asan Medical Center, University of Ulsan College of Medicine, Seoul, Republic of Korea

**Keywords:** Oncogenes, Polycystic kidney disease

## Abstract

Ubiquitination serves a critical role in regulating both inflammatory responses and kidney injury. Among inherited renal disorders, autosomal dominant polycystic kidney disease (ADPKD) has demonstrated associations with disrupted ubiquitin signaling that exacerbates inflammation and cyst progression. In this study, we demonstrate that the E3 ligase Pellino1 (Peli1) acts as an essential contributor to the pathogenesis of ADPKD amid inflammatory conditions. In individuals with clear cell renal cell carcinoma (ccRCC), Peli1 exhibits markedly elevated expression, and this upregulation is associated with adverse clinical outcomes. Additionally, we find that various TLR stimulations in renal tubular cells induce increased Peli1 expression, which is also elevated in samples from ADPKD patients. Using doxycycline-inducible Peli1-transgenic mice, we establish that Peli1 overexpression leads to impaired renal function and facilitates cyst formation. On a mechanistic level, elevated Peli1 promotes cystic epithelial cell proliferation by activating mTOR signaling, accomplished through the stabilization of S6K1. In summary, our data indicate that TLR-driven upregulation of Peli1 facilitates renal cyst growth via S6K1 stabilization. These results reveal a novel mechanistic link between PKD and ccRCC.

**Figure:**
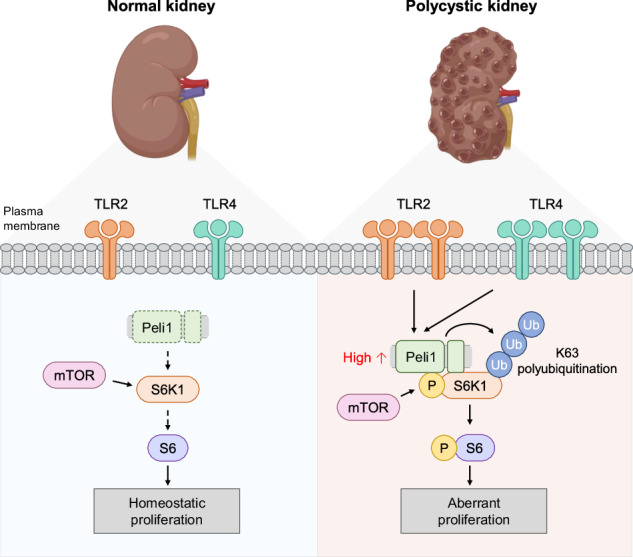
A schematic model is proposed to describe the role of Peli1 in the development of polycystic kidney diseases. Normal signaling pathways (Left) and Peli1-mediated signaling pathways in polycystic kidney disease (Right). The illustration outlines the cascade from TLR stimulation to Peli1-dependent K63 ubiquitination of S6K1 and subsequent proliferation in renal tubular epithelial cells. This figure was generated using BioRender.com.

A schematic model is proposed to describe the role of Peli1 in the development of polycystic kidney diseases. Normal signaling pathways (Left) and Peli1-mediated signaling pathways in polycystic kidney disease (Right). The illustration outlines the cascade from TLR stimulation to Peli1-dependent K63 ubiquitination of S6K1 and subsequent proliferation in renal tubular epithelial cells. This figure was generated using BioRender.com.

## Introduction

Autosomal dominant polycystic kidney disease (ADPKD) is a frequent monogenic renal disorder, exhibiting a prevalence of about 1 in 1000 individuals and characterized by the development of prominent, fluid-filled cysts originating from renal tubules [1]. ADPKD is predominantly caused by inherited mutations in *PKD1* or *PKD2* [2, 3], where truncating or inactivating variants within the renal tubules contribute to disease severity. As cysts grow, they exert pressure on nearby nephrons, diminish parenchymal renal function, and ultimately lead to chronic kidney disease or end-stage renal failure [4]. Nearly 50% of individuals with ADPKD eventually develop end-stage renal disease, requiring interventions such as kidney transplantation or dialysis.

One of the most significant comorbidities associated with ADPKD is the development of renal cell carcinoma (RCC) [5]. RCC represents the second most common cause of death among patients with urological cancers and is responsible for more than 90% of kidney cancers [6]. Multiple studies have documented an elevated incidence of RCC in individuals diagnosed with ADPKD [7]. RCC and ADPKD exhibit overlapping characteristics, including the presence of cysts, neoplastic cell populations, and involvement of specific renal tubules [8]. Among the underlying molecular mechanisms in kidney disorders, ubiquitination has a crucial role in regulating inflammation and mediating kidney injury [9]. In the context of ADPKD, impairments in ubiquitin signaling lead to enhanced inflammation and facilitate cyst development [10, 11]. In particular, aberrant regulation of E3 ubiquitin ligases may alter protein stability or functional activity. As a result, this affects cytokine synthesis, abnormal cell proliferation, and fibrotic tissue changes characteristic of PKD pathology [9]. Nevertheless, the specific mechanisms by which ubiquitination modulates the inflammatory response in PKD are not yet completely understood.

Among inflammatory mediators, Toll-like receptors (TLRs) have been widely implicated in renal inflammation, kidney injury, and fibrosis, ultimately influencing both the inhibition and progression of acute and chronic kidney diseases [12, 13]. These receptors act as pathogen recognition receptors, identifying conserved structural motifs such as pathogen-associated molecular patterns (PAMPs) and damage-associated molecular patterns (DAMPs) [14]. Following activation by either exogenous or endogenous ligands, TLRs—which are expressed on both immune and non-immune cells, including renal tubular epithelial cells (TECs) [15, 16]—can initiate the nuclear factor-κB (NF-κB) signaling pathway [17]. Activation of this pathway elicits the production of multiple pro-inflammatory cytokines and chemokines, which contribute to organ injury [18]. Alterations in TLR expression, including TLR1/2/3/4/6 and TLR7, have been observed in TECs in association with the renal pathology of several kidney diseases [12, 19, 20]. Importantly, elevated gene expression of *TLR2* and *TLR4* has been associated with the accelerated progression of chronic kidney disease, including ADPKD [21].

Peli1 is an ubiquitin E3 ligase characterized by a C3HC4 RING-like motif within its C-terminal domain, and is necessary for mediating inflammatory diseases and/or cancer through the regulation of multiple receptor signaling pathways [22, 23]. Peli1 plays a central role in the development of inflammation and autoimmunity via its regulation of TLR and T cell receptor signaling [24]. In response to TLR3 and TLR4 ligands, Peli1 deficiency results in decreased production of pro-inflammatory cytokines [25]. Furthermore, Peli1 impairs the metabolic reprogramming of CD8^+^ T cells by influencing the function of mammalian target of rapamycin (mTOR) complex 1 [26]. Although post-transcriptional modifications via phosphorylation have been studied, relatively few investigations—especially those focusing on ubiquitination—have examined the regulatory effect of Peli1 in modulating signaling cascades that contribute to kidney disease via receptor pathways.

Activation of mTOR signaling, primarily discussed within the context of proliferative mechanisms, is implicated in the development of renal cysts resulting from defective ubiquitination [10, 27]. Moreover, clinical research is ongoing to elucidate the precise functions of mTOR signaling in enhancing RCC treatment [28, 29]. The purpose of this study was to determine whether TLR-driven Peli1 regulates renal cyst progression through the activation of mTOR.

In this study, we first identified that data from patients with clear cell renal cell carcinoma (ccRCC) reveal a significant association between Peli1 expression levels and both prognosis and overall survival. Additionally, renal tubular cells demonstrated elevated Peli1 expression in response to TLR activation, particularly TLR2 and TLR4, and *TLR2*, *TLR4*, and *Peli1* exhibited higher expression in individuals with ADPKD. Peli1 overexpression led to reduced renal function, formation of renal cysts, and increased proliferation of TECs, all features characteristic of ADPKD. Mechanistically, Peli1 directly binds to S6K1, activating the mTOR signaling pathway, which is strongly implicated in the development of ADPKD and RCC [30–32]. Collectively, our findings indicate that Peli1 is a crucial regulator of renal cyst proliferation via the mTOR pathway and serves as a promising therapeutic target for ADPKD.

## Methods

### Ethics approval and consent to participate

All animal experiments were performed in accordance with the guidelines of the Institutional Animal Care and Use Committee (IACUC 2021-11-27-3, IACUC 2022-10-34-1, IACUC 2023-01-08-1, IACUC 2023-10-16-1, IACUC 2024-03-60-1, IACUC 2024-10-65-2, and IACUC 2025-10-40-1) of Sungkyunkwan University School of Medicine (SKKU-SOM). The SKKU-SOM is accredited by the Association for Assessment and Accreditation of Laboratory Animal Care International and adheres to the Institute of Laboratory Animal Resources guidelines. Human tissue samples and associated clinical data were collected in accordance with relevant guidelines and regulations. The study protocol was approved by the Institutional Review Board (IRB) of Asan Medical Center (IRB No. 2012-0788). The requirement for written informed consent was waived by the IRB due to the retrospective nature of the study and the use of anonymized archival specimens. This study did not include identifiable images of human participants; therefore, consent for publication was not applicable.

### Animal studies

Doxycycline-inducible human Peli1-transgenic mice (rtTA-Peli1) [24] were used in this study. pTRE TetO Myc-Peli1-transgenic mice were obtained from Ho Lee (National Cancer Center, South Korea). To create doxycycline-inducible human PELI1-transgenic mice (rtTA-Peli1), pTRE TetO Myc-Peli1-transgenic mice were purchased from The Jackson Laboratory and crossed with R26-M2rtTA mice (B6.Cg-Gt(ROSA)26Sortm1(rtTA*M2)Jae/J). These animals were kept under specific pathogen-free conditions. All experiments involved mice homozygous for both the R26-M2rtTA and human PELI1 transgenes. To induce expression of the human PELI1 transgene, 4-week-old rtTA-Peli1 mice (C57BL/6) were randomly given drinking water supplemented with 2 mg/mL doxycycline (Sigma) and 5% sucrose (Sigma), which was shielded from light and replaced every 3 days.

### Patients and samples

Patients with recurrent or metastatic RCC administered with vascular endothelial growth factor receptor-tyrosine kinase inhibitors (TKIs) at Asan Medical Center, Seoul, Republic of Korea, between 1997 and 2013 were identified. Of the 533 patients meeting these criteria, 75 cases with available tissue samples, all of whom had undergone radical nephrectomy followed by TKI therapy, were randomly selected for Peli1 immunohistochemical staining. A tissue microarray was constructed from two representative cores, each 1.5 mm in diameter, derived from FFPE tumor tissues and used for immunohistochemical staining. Immunohistochemistry was conducted using an automated system (BenchMark XT, Ventana Medical Systems, Tucson, AZ) following the manufacturer’s guidelines.

### Urinary and kidney function analyses

For proteinuria analysis, 20 µl spot urine samples from each mouse at the time of sacrifice were mixed with SDS-sample loading buffer and subjected to 10% sodium dodecyl sulfate-polyacrylamide gel electrophoresis (SDS-PAGE). Gels were stained with SimplyBlueTM SafeStain (Invitrogen, Carlsbad, CA) for 1 h, followed by a double-distilled water (ddH_2_O) wash for 1 h. Albumin (Gendepot, A0100-010) served as the control. BUN levels were assessed following the manufacturer’s protocol (QuantiChrom Urea Assay Kit; BioAssay Systems, 501079333).

### Histological analysis and immunohistochemistry (IHC) staining

Kidney tissues were fixed in 10% formalin solution, embedded in paraffin, sectioned into 5 µm slices, and stained using hematoxylin and eosin. All procedures adhered to standard histological methods. Slides were examined using a MoticEasyScan Pro6 (Motic) Scanner, with images reviewed using Aperio ImageScope software v.12.4.3.5008 (Leica Biosystems). Tissue analyses were conducted using ImageScopex64 based on sectional H&E staining images from a single mouse.

For immunofluorescence analysis, paraffin-embedded renal sections were rehydrated through a xylene and decreasing ethanol series, washed in Tris-buffered saline, and boiled in 10 mM sodium citrate buffer (pH 6) for 15 min. The sections were cooled, washed with TBS-buffered saline containing 0.1% tween 20 (TBS-T), and then blocked with 5% goat serum in PBS at 37 °C for 45 min. After washing with TBS-T for 5 min twice, sections were incubated with fluorescence-labeled antibodies diluted in blocking buffer at 37 °C for 1 h. The antibodies used included fluorescence-labeled lotus tetragonolobus lectin (LTL) and dolichos biflorus agglutinin (DBA) from Vector Laboratories, and THP and PCNA from Santa Cruz Biotechnology Inc. Following another wash with TBS-T, sections were postfixed with 10% neutral-buffered formalin for 10 min, washed with TBS-T, and mounted with ProLong Gold Antifade Mounting Medium with DAPI (Thermo Fisher Scientific). For immunohistochemistry, the sections were deparaffinized, treated with citrate buffer for antigen retrieval, stained with anti-P-S6 (Ser240/244) (Cell Signaling, #2215) and anti-Peli1 (Santa Cruz, #sc-271), and incubated using Vectastain Elite ABC kit (Vector Laboratories). The peroxidase activity was visualized using a 3,3′-diaminobenzidine (DAB) substrate kit (Vector Laboratories), and tissues were counterstained with Harris hematoxylin (BBC Biochemical).

### Wound healing assay

After cell transfection at 37 °C with 5% CO_2_ for 24 h, a wound was created using a 200 μl pipette tip on the cell monolayer, and photographs were taken at the appropriate times to estimate the area occupied by migratory cells.

### Immunoblotting

Protein lysates were prepared from cells or tissue using RIPA buffer (150 mM NaCl, 20 mM pH 7.4 Tris-HCl, 0.1% SDS, 1% Triton X-100, 1 mM EDTA, 1 mM Na_3_VO_4_, Phenylmethylsulfonyl fluoride (PMSF), and 1X protease inhibitor cocktail (PIC)). Kidney tissues were homogenized with a Polytron Homogenizer PT 1300D (Switzerland, Kinematica AG) in RIPA buffer for tissue lysate preparation. Cells were homogenized in cold RIPA buffer through a syringe tip. Protein lysates were quantified by the Bradford assay and separated by SDS-PAGE. Proteins containing gels were transferred onto nitrocellulose (NC) membranes. The membranes were then blocked with TBS-T containing 5% skim milk for 1 h at room temperature (RT) and incubated with primary antibodies in 5% BSA or 5% skim milk for overnight at 4 °C or 3 h at RT. After washing, they were probed with HRP-conjugated anti-Rabbit IgG-HRP (GenDEPOT, #SA002-500) and anti-Mouse IgG-HRP (GenDEPOT, #SA001-500) antibodies diluted 5% skim milk for 2 h at RT, and subsequently washed for 1 h with TBS-T. Bands were detected using ECL solution (AbFrontier) and developed on Medical X-ray film blue (AGFA). All antibodies are listed in Supplementary Table 1.

### Plasmid construction

The complete cDNA sequence of human Peli1 proteins was obtained using oligo-dT primers. Both Peli1 full-length and Peli1 ΔC (1–275) were subcloned into Myc-tagged and GFP-tagged plasmids, respectively. Peli1 ΔC comprised 280 N-terminal amino acids and lacked the C-terminal RING domain. GFP-tagged Peli1 RING mutants were generated using a QuickChange site-directed mutagenesis kit (Stratagene). The human codon sequences of genes encoding S6K1 were constructed via molecular cloning by PCR and then subcloned into a FLAG-tagged expression plasmid.

### Cell culture and transient transfection

The 786-O cells (ATCC, CRL-1932) and HK-2 (ATCC, CRL-2,190) were cultured in RPMI containing 1% penicillin/streptomycin antibiotics and 10% FBS (Gibco). Cells were maintained at 37 °C in 90% humidity and a humidified atmosphere of 5% CO_2_. For transfection, cells were plated onto 6-well tissue culture plates 24 h before transfection. Transient transfection of the Myc-Peli1 or control Myc plasmid was performed using Lipofectamine 2000 (Invitrogen) as per the manufacturer’s protocol.

### Lentivirus production and lentiviral transduction

The lentiviral transfer vector DNA, in conjunction with psPAX2 packaging and pMD2. The G envelope plasmid DNA was mixed at a 4:3:1 ratio and used to transfect 293T cells (ATCC, CRL-3216) to produce a virus. Sixteen hours after transfection, the medium was replaced with DMEM supplemented with 10% FBS unless indicated otherwise, and incubated at 5% CO_2_ before the initial viral supernatant collection. Supernatants were collected after 96 h. The harvested medium was cleared by centrifugation at 1500 rpm for 5 min at 4 °C and then filtered through a 0.45 μM pore PVDF Millex-HV filter (Millipore). Seed HK-2 cells were seeded in a 24-well plate to 50% confluency prior to transduction. HK-2 cells were incubated for 18 h with culture media containing lentiviral particles and polybrene. To establish a stable cell line, the cells were maintained in complete medium containing puromycin.

### Isolation and culture of renal tubular epithelial cells (TECs)

Primary renal tubular epithelial cells were isolated from rtTA and rtTA-Peli1 mice aged 4 weeks. [33] For Peli1 induction in cultured renal tubular epithelial cells, doxycycline was added to the media at a concentration of 2 μg/ml.

### In vitro binding and immunoprecipitation assays

For pull-down assays, 1 mg of fusion protein was adsorbed onto A/G-Sepharose beads (Amersham Biosciences) and rotated with 1 mg of whole-cell extracts from 293T cells for 12 h. Transfected 293T cells were lysed with IP buffer [150 mmol/L NaCl (Sigma), 20 mmol/L Tri-HCl (Sigma), 5 mmol/L EDTA (GenDEPOT), 1% Triton X-100 (Sigma), 1 mmol/L phenylmethanesulfonylfluoride (Sigma), 10 mmol/L NaF (Sigma), 1 mmol/L Na_3_VO_4_ (Sigma), and 1 mmol/L DTT (Sigma) supplemented with a PIC (GenDEPOT; 50 mmol/L PMSF, 10 mmol/L pepstatin A, 20 mmol/L leupeptin, 100 mmol/L benzamidine, and 50 mmol/L bestatin)] and incubated at 4 °C for 30 min. The lysates were then centrifuged at 13,000 rpm for 30 min, and the insoluble fraction was discarded. The bound proteins were resolved by SDS-PAGE and analyzed by immunoblotting as described below.

For immunoprecipitation assays, HK-2 cells expressing Flag-S6K1 with or without GFP-Peli1 were lysed as described above using IP buffer. Lysates (0.5 mg) were incubated with an anti-GFP antibody (1 mg/1 mg) or normal IgG (control, 1 mg/1 mg) and then incubated with protein A/G agarose beads for 12 h. After incubation, the cells were pelleted, washed three times in immunoprecipitation buffer, and analyzed by immunoblotting as described below.

### In vivo ubiquitination assay

293T cells were transfected with an expression plasmid encoding Myc-Peli1, Myc-Peli1 ΔC; GFP, GFP-tagged Peli1 WT, H313A, C336A mutants; Flag-S6K1; HA-Ub in specific combinations. At 48 h post-transfection, cells were divided into two aliquots. One aliquot underwent conventional immunoblotting. The other was used for immunoprecipitation with anti-Flag antibodies. Immunoprecipitates were washed three times with IP buffer, and bound proteins were subsequently immunoblotted with the indicated antibodies.

### Sirius red staining

Mouse kidney paraffin tissue blocks were prepared using standard methods and sectioned for consecutive levels. Sections were deparaffinized, rehydrated, and stained with 0.1% Sirius Red in aqueous saturated picric acid for 1 h. Following this, sections were washed in acidified water (0.5% HCL), dehydrated, and mounted.

### Dual X-ray absorptiometry (DEXA)

Mice underwent bone mineral density (BMD) scans at 0-, 4-, 8-, and 12-week intervals after doxycycline treatment using a dual X-ray absorptiometry (DEXA) Bone Densitometer for small animals. Prior to each scan, mice were weighed and anesthetized to ensure immobilization during the 5-min scan. Post-scan, mice were allowed to recover from anesthesia before being returned to their cages. Whole-body BMD was quantified.

### Quantitative real-time PCR (qRT-PCR)

Total RNA was extracted from kidneys using Qiazol (QIAGEN) or the RNeasy Mini Kit (QIAGEN). The cDNAs were synthesized using a High Capacity cDNA Reverse Transcription Kit (Applied Biosystems). For PCR amplification, the cDNAs were performed with a Power SYBR Green Chemistry (Applied Biosystems). All procedures followed the respective manufacturer’s protocols. Each sample was processed in triplicate. Relative expression levels were normalized against *Gapdh* as a control, and determined using the comparative Ct (2^−∆∆Ct^) method. All primer sequences are listed in Supplementary Table 2.

### Statistical analysis

Data are presented as means ± SEMs from at least three independent experiments. All data were analyzed using the GraphPad Prism 4.5 software package (GraphPad Software). Statistical significance was analyzed using Student’s *t*-test or two-way ANOVA. A Dunnett’s post-hoc test was used to test for significant differences following two-way ANOVA. *p*-values of less than 0.05 (<0.05) were regarded as statistically significant for all tests. The mRNA levels of Peli1 in normal or ccRCC tissues were analyzed using the Oncomine dataset. Kaplan–Meier plots demonstrating cancer -specific survival and progression -free survival of ccRCC patients, stratified by Peli1 mRNA expression levels, were analyzed using Kaplan–Meier survival datasets, and the differences between groups were compared using the log-rank test.

## Results

### Peli1 overexpression correlates with adverse outcomes in ccRCC patients

**Fig. 1:**
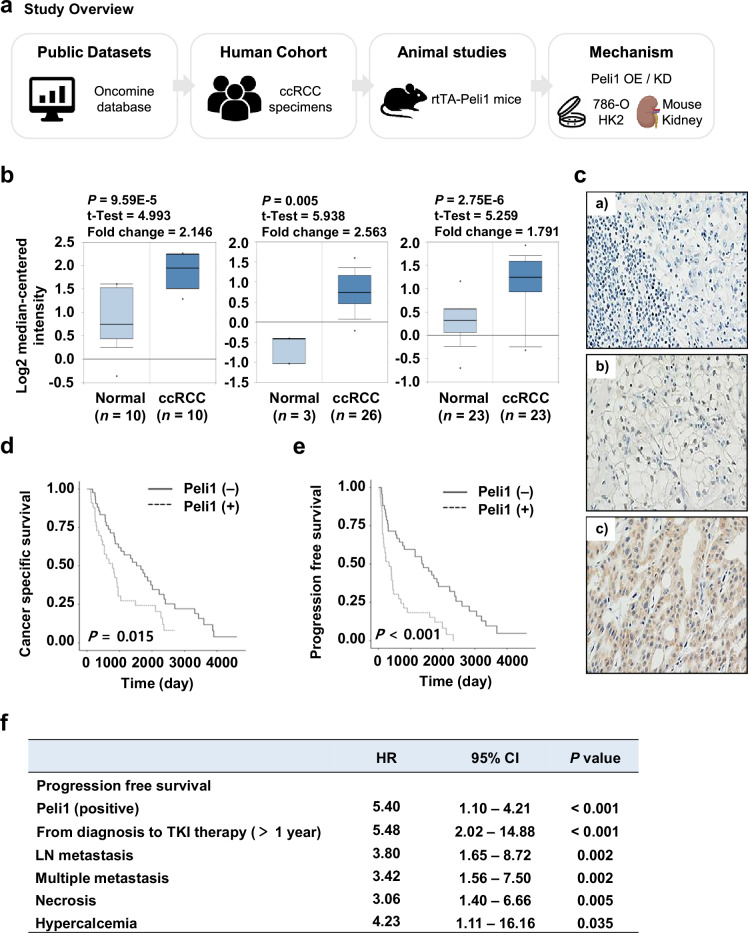
Elevated Peli1 expression correlates with poor prognosis in ccRCC patients. **a** Study overview diagram. OE overexpression, KD knockdown. **b** Comparison of Peli1 mRNA expression in normal and ccRCC kidney tissues. Box plots derived from gene expression data in ONCOMINE comparing the expression of Peli1 in normal and ccRCC kidney tissues. Data are presented as means ± SEMs. Statistical significance was determined by the two-sided Student’s *t*-test. **c** Representative evaluation of Peli1 expression in ccRCC specimens by immunohistochemistry. (**a**, negative; **b**, mild; **c**, strong immunoactivity). Kaplan–Meier survival analysis for cancer-specific survival (**d**) and progression-free survival (**e**) according to Peli1 expression. ccRCC patients with positive Peli1 expression (*n* = 33) exhibited significantly worse overall survival compared to those with negative expression (*n* = 42). **f** Multivariate Cox-regression survival analysis. Kaplan–Meier survival curves (**d**, **e**) were evaluated by the two-sided log-rank test to assess statistical significance.

To clarify the systematic approach of this study, we first outlined the sequential investigations conducted, which included the analysis of public datasets, evaluation of human cohorts, experimentation using mouse models, and a series of mechanistic studies (Fig. 1a). Elevated Peli1 expression has been associated with tumor progression and poor prognosis across various cancers, such as B cell lymphoma and breast cancer, affecting tumor characteristics, immune responses, and the efficacy of combination therapies [23, 34]. To investigate the relationship between Peli1 and RCC development, we examined its expression in ccRCC tissues, a subtype of RCC, utilizing gene expression profiles from the ONCOMINE database. Our analysis revealed that Peli1 expression was markedly increased in ccRCC samples compared to normal renal tissue (Fig. 1b). To corroborate these findings, 75 ccRCC kidney tissue samples were obtained from patients who had undergone radical nephrectomy followed by VEGF TKI treatment, and Peli1 levels were assessed by IHC staining. There was a notable rise in both the intensity and extent of Peli1 immunoreactivity across a range of ccRCC patients’ tissues (Fig. 1c). Next, we performed a survival analysis on patients with available follow-up data to evaluate whether Peli1 expression relates to clinical outcomes. Kaplan–Meier survival curves demonstrated that ccRCC patients with detectable Peli1 expression had reduced overall survival compared to those lacking such expression (Fig. 1d, e). Furthermore, multivariate Cox-regression analysis established Peli1 expression as an independent predictor of poor progression-free survival (Fig. 1f). Together, these results demonstrate that elevated Peli1 expression is associated with unfavorable prognosis in ccRCC patients.

### Excessive Peli1 expression in the kidney has been found to contribute to renal failure

**Fig. 2:**
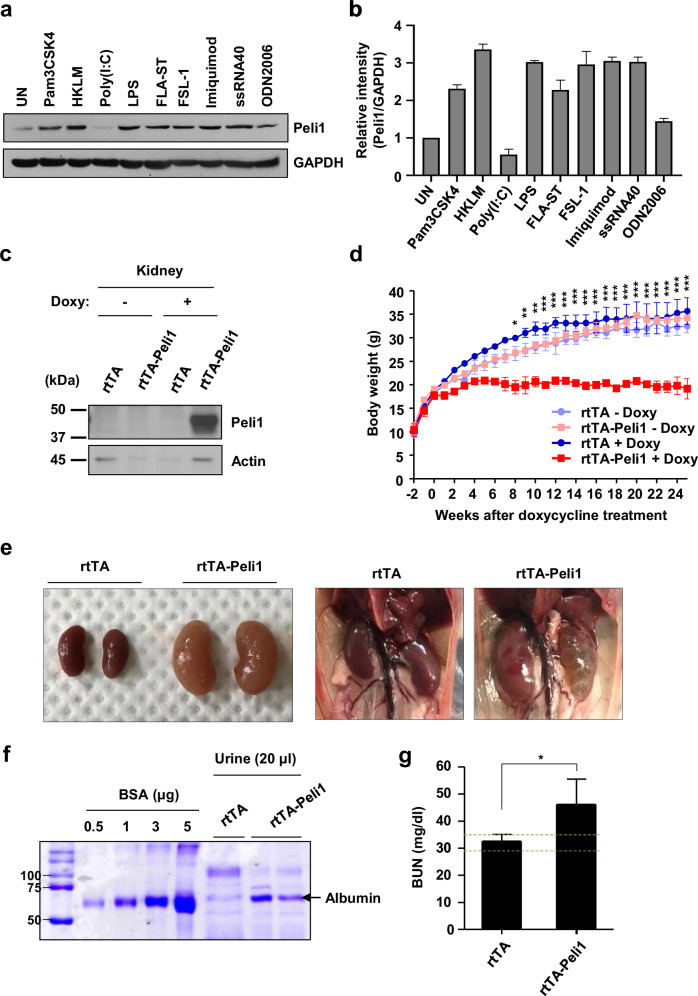
Peli1 overexpression reduces renal function. **a** Immunoblots of 786-O cells stimulated for 6 h with respective TLR agonists are shown. (Pam3CSK4, TLR1/2 ligand; HKLM, TLR2 ligand; poly (I:C), TLR3 ligand; LPS, TLR4 ligand; ST-FLA, TLR5 ligand; FSL-1, TLR2/6 ligand; Imiquimod, TLR7 ligand; ssRNA40, TLR8 ligand; ODN2006, TLR9 ligand). **b** Bar graph illustrating the relative intensity of Peli1. Band intensity was quantified by densitometry with ImageJ software, and normalized to GAPDH within each sample (*n* = 3). **c** Immunoblotting of kidney lysates from rtTA and rtTA-Peli1 mice using the specified antibodies revealed markedly elevated Peli1 protein levels in kidney tissues. **d** Doxycycline -treated rtTA-Peli1 mice showed growth delay. *n* = 10 for each genotype at the start of the study. **e** Shown are representative kidney images from rtTA and rtTA-Peli1 mice after 24 weeks of doxycycline treatment. **f** Coomassie blue staining of urinary proteins separated by gel electrophoresis was performed at 24 weeks post-Doxycycline treatment. **g** Plasma blood urea nitrogen (BUN) concentrations were measured to assess kidney function at 12 weeks after doxycycline treatment (*n* = 3). The green grid line denotes the reference interval of BUN at 12 weeks after doxycycline treatment. Data are expressed as means ± SEMs. Shown are representative blots from three independent replicates. Two-way ANOVA was used for all statistical comparisons. *P < 0.05; **P < 0.01; ***P < 0.001.

RCC is reported to occur more frequently in ADPKD patients than in healthy individuals [35, 36]. TLR signaling is capable of eliciting innate inflammatory responses that result in kidney injury [18]. It has been reported that various TLRs are expressed in renal tubular epithelial cells [15, 16]. Particularly, the upregulation of TLR2 and TLR4 is linked to the accelerated progression of ADPKD [21]. To assess Peli1 expression in renal tubular epithelial cells under TLR stimulation, we examined Peli1 expression in the 786-O human kidney tubular cell line. Peli1 protein expression increased following stimulation with most agonists, especially TLR2/4/7/8 in 786-O (Fig. 2a, b). We then evaluated TLR and Peli1 expression in ADPKD by performing RNA profiling using publicly available database analysis [37]. We found that the expression levels of *TLR2*, *TLR4*, and *Peli1* were elevated in cystic kidneys from human ADPKD (Supplementary Fig. 1a), indicating that dysregulation of TLRs and Peli1 is involved in the pathogenesis of ADPKD.

Given the marked upregulation of Peli1 in ADPKD, we established doxycycline-inducible human Peli1-transgenic mice (referred to as rtTA-Peli1 mice) to evaluate the effects of Peli1 overexpression [24]. We confirmed that Peli1 was overexpressed across multiple organs, including the kidneys (Fig. 2c and Supplementary Fig. 2a) [24]. Notably, doxycycline-treated rtTA-Peli1 mice exhibited reduced body size and a distended abdomen in contrast to control rtTA mice (Supplementary Fig. 2b). Assessment of body weight over time in rtTA/rtTA-Peli1 mice demonstrated divergence following 8 weeks of doxycycline administration (Fig. 2d). Subsequent examinations revealed that both kidneys in rtTA-Peli1 mice were markedly enlarged at 24 weeks after doxycycline exposure (Fig. 2e). Urine samples collected from rtTA and rtTA-Peli1 mice were analyzed by SDS-PAGE with Coomassie blue staining, which identified significant proteinuria in the rtTA-Peli1 group 24 weeks after doxycycline administration (Fig. 2f). In comparison to rtTA control mice, rtTA-Peli1 mice displayed substantially elevated blood urea nitrogen (BUN) levels following 12-week doxycycline induction (Fig. 2g). Collectively, these findings demonstrate that overexpression of Peli1 impairs renal function.

### Peli1 overexpression drives polycystic kidney disease development and augments NF-κB-mediated renal inflammation

**Fig. 3:**
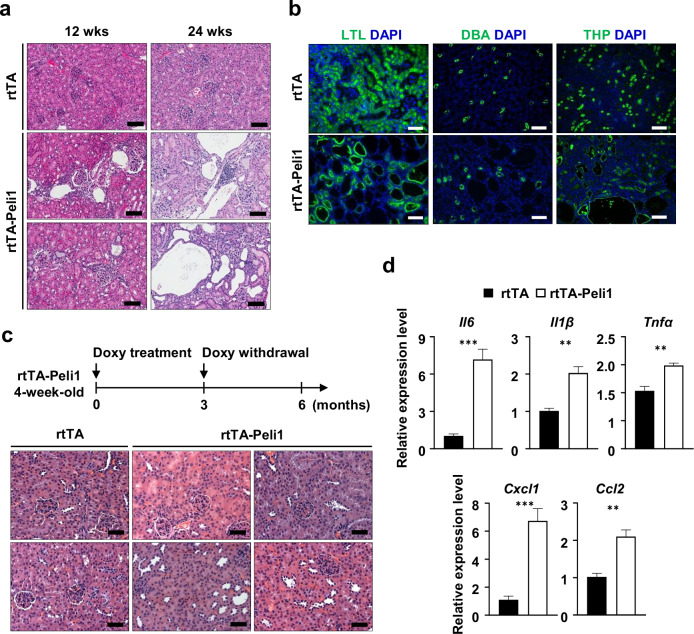
Peli1 overexpression promotes the formation of kidney cysts and NF-κB-dependent renal inflammation. **a** H&E-stained kidney sections from each mouse group treated with doxycycline for 12 or 24 weeks are presented. Scale bars represent 200 μm. **b** Kidney sections from rtTA and rtTA-Peli1 mice at 24 weeks post-Doxycycline treatment were stained with LTL (a proximal tubule marker), DBA (a collecting duct marker), and THP (a marker of loops of Henle). Scale bars represent 50 μm. **c** Schematic of the experimental protocol using doxycycline-inducible transgenic mice subjected to different doxycycline administration schedules. Representative H&E-stained kidney sections from rtTA and rtTA-Peli1 mice after doxycycline withdrawal at the indicated time points. Scale bars represent 50 μm. **d** The expression of key genes involved in NF-κB-mediated cytokine production was examined in kidney tissue by qRT-PCR following 12 weeks of doxycycline administration (*n* = 4). Results were normalized to *Gapdh*. Data are expressed as means ± SEMs. Student’s *t*-test was used to assess statistical significance. **P < 0.01; ***P < 0.001.

To analyze renal pathology in rtTA-Peli1 mice, kidneys were collected from both rtTA and rtTA-Peli1 mice. Kidneys from rtTA mice displayed normal histological architecture. In contrast, beginning 12 weeks after doxycycline administration, rtTA-Peli1 mouse kidneys exhibited prominent diffuse tubular dilatation, attenuation accompanied by focal lymphocytic infiltration, tubular atrophy, and increased interstitial fibrosis, while the glomeruli largely appeared unaffected (Fig. 3a). Pronounced Peli1 overexpression was detected in the cyst-lining cells, which exhibited a shift from cuboidal to squamous morphology (Supplementary Fig. 2a). At 24 weeks post-doxycycline, there was a marked increase in both the size and number of renal cysts, predominantly observed in the cortex, whereas the medulla was largely spared (Supplementary Fig. 3a, b). Extensive cystic changes corresponded with a notable rise in fibrosis (Supplementary Fig. 3c). Immunostaining for specific renal tubular markers demonstrated that these cysts originated primarily from the proximal tubules and the loops of Henle (Fig. 3b). We further evaluated whether cessation of doxycycline could improve established renal cysts in rtTA-Peli1 mice (Fig. 3c). Remarkably, kidney pathology was largely ameliorated upon normalization of Peli1 expression levels (Fig. 3c). Overall, these results support that Peli1 overexpression alone drives renal cyst formation in mice.

To gain further insight into the inflammatory mechanisms underlying Peli1-induced cystogenesis, we assessed the expression of canonical NF-κB-responsive cytokines in kidneys from doxycycline-treated rtTA-Peli1 mice. qRT-PCR analysis demonstrated upregulation of *Il6*, *Il1β*, *Tnfα*, *Cxcl1*, and *Ccl2*, in comparison to rtTA control mice (Fig. 3d), indicating activation of the NF-κB pathway in cystic kidneys [38]. Collectively, these observations indicate that Peli1 acts as a major TLR-inducible enhancer of NF-κB-dependent cytokines, thereby linking innate immune pathways to cyst progression.

Alterations in hormone and growth factor secretion are common complications observed in chronic renal failure [39]. Furthermore, individuals with polycystic kidney disease frequently present with growth impairment, skeletal abnormalities, and metabolic disturbances [40–42]. We assessed the BMD of rtTA-Peli1 mice receiving doxycycline for 4, 8, and 12 weeks, as well as that of their rtTA littermates, utilizing DEXA scans. Notably, the rtTA-Peli1 mice demonstrated lower BMD compared to the control rtTA group (Supplementary Fig. 4a, b). Thus, Peli1-transgenic mice manifested polycystic kidney disease with related complications similar to those observed in humans.

### Peli1 facilitates the proliferation and migration of cystic renal epithelial cells

**Fig. 4:**
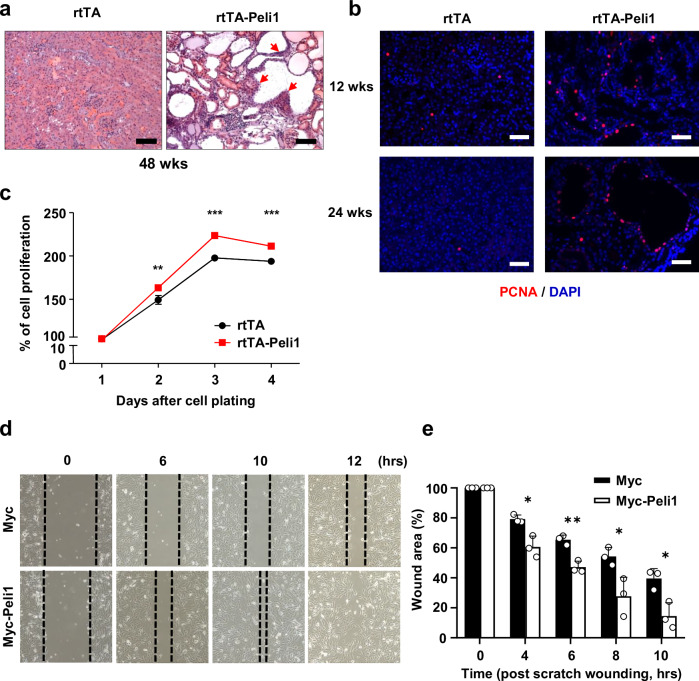
Peli1 overexpression augments the proliferation and migration of renal tubular epithelial cells. **a** H&E-stained sections of kidneys from mice in each group were obtained after treatment with doxycycline for 48 weeks. Scale bars, 200 μm. **b** Immunofluorescence staining was performed to detect PCNA expression in representative kidney sections from rtTA and rtTA-Peli1 mice treated with doxycycline for 12 and 24 weeks. Scale bars, 50 μm. **c** Primary renal tubular epithelial cells were isolated from rtTA and rtTA-Peli1 mice that had not received doxycycline and subsequently treated with 2 μg/ml doxycycline after plating. The proliferation rate of rtTA-Peli1 cells was significantly increased (*n* = 3). Statistical significance was assessed by two-way ANOVA. **d** Cell migration of 786-O cells transfected with Myc-Peli1 or Myc control was examined using wound healing assays, and representative images were taken at 0, 6, 10, and 12 h. **e** The percentage of wound area at the indicated time points was calculated relative to the initial area at 0 h (*n* = 3). Data for (**c**, **e**) are expressed as means ± SEMs. Statistical analysis was performed using Student’s *t*-test. *P < 0.05; **P < 0.01; ***P < 0.001.

Patients with PKD are recognized as being at higher risk of developing RCC lesions [43]. To determine the propensity for tumorigenesis, we examined the kidneys of mice administered doxycycline for 1 year. Notably, we identified atypical proliferation of tubular epithelial cells in the kidneys of doxycycline-treated rtTA-Peli1 mice (Fig. 4a). This tubular epithelial hyperplasia eventually progressed to tumorigenesis following a prolonged latency period [44, 45]. To investigate whether heightened cell proliferation may play a role in promoting renal cyst growth, we assessed whether Peli1 overexpression accelerates cyst expansion through increased cell proliferation. We performed immunostaining of kidney sections with anti-PCNA antibodies to detect proliferative cells. A pronounced increase in proliferation of renal tubular epithelial cells was observed in doxycycline-treated rtTA-Peli1 mouse kidneys at both 12 and 24 weeks compared to control mice (Fig. 4b). Additionally, reducing of Peli1 led to a substantial decline in the population of PCNA-positive cells within the kidney (Supplementary Fig. 5a). To clarify whether the proliferation of tubular epithelial cells stems from external stimuli (PAMPs, DAMPs, cytokines, and chemokines) [46] or from cell-intrinsic Peli1 overexpression, we isolated primary renal tubular epithelial cells from the kidneys of rtTA and rtTA-Peli1 mice. Cell proliferation was significantly higher in renal tubular epithelial cells derived from rtTA-Peli1 mice relative to those from rtTA mice (Fig. 4c). Furthermore, we assessed the effect of Peli1 overexpression on cancer cell motility using wound healing assays in the 786-O renal adenocarcinoma cell line. Importantly, Peli1 overexpression resulted in a substantial increase in migratory capacity in 786-O cells compared to the controls (Fig. 4d, e and Supplementary Fig. 5b). Collectively, these results indicate that Peli1 promotes cyst growth by facilitating both proliferation and migration of cystic epithelial cells.

### Aberrant Peli1 expression activates mTOR signaling via stabilization of S6K1

**Fig. 5:**
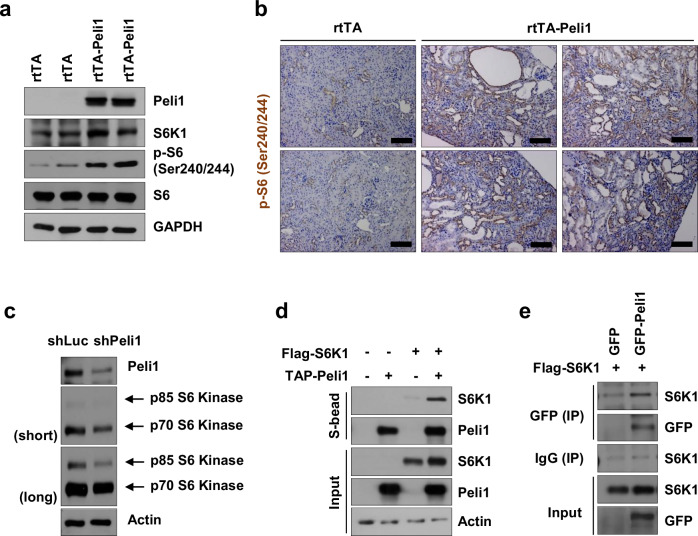
Peli1 interacts with the S6K1 protein. **a** Immunoblot analyses were conducted for the indicated proteins in kidney tissues from rtTA and rtTA-Peli1 mice following 12 weeks of doxycycline administration. **b** Immunohistochemical analysis was carried out for p-S6 (Ser240/244) expression in kidneys at 12 weeks after doxycycline treatment in rtTA and rtTA-Peli1 mice. Scale bars, 50 μm. **c** Western blotting was used to measure Peli1 and S6K1 protein levels after infection with lentiviral particles containing Peli1 shRNA in HK-2 cells. **d** Pull-down assays were conducted on 293T cells co-expressing TAP-Peli1 and flag-S6K1; TAP-Peli1 was precipitated with S-beads, and S6K1 co-precipitation was detected by immunoblotting using an anti-S6K1 antibody. **e** Co-immunoprecipitation was performed for GFP-Peli1 and Flag-S6K1 co-expressed in HK-2 cells. GFP-Peli1 was immunoprecipitated with an anti-GFP antibody, and detection of co-precipitated Flag-S6K1 was done by immunoblotting using the same antibody.

To clarify the mechanism by which Peli1 promotes the proliferation and migration of cystic renal epithelial cells, we assessed the expression profiles of key markers associated with signaling pathways involved in cyst epithelial proliferation within kidney tissues from rtTA and rtTA-Peli1 mice 12 weeks after doxycycline administration (Supplementary Fig. 6). Peli1 overexpression did not change the expression or phosphorylation of c-Myc, EGFR, AKT, AMPK, or MEK1/2. ERK activation most likely results from secondary stimulation via inflammatory cytokines rather than a direct action of Peli1 on the MAPK pathway. We next evaluated mTOR signaling by analyzing cell lysates obtained from whole kidneys of rtTA and rtTA-Peli1 mice (Fig. 5a). mTOR signaling is recognized for its role in promoting cell growth and proliferation across multiple cell types [47], and aberrant mTOR pathway activation has been documented in PKD [32, 48, 49]. Previous reports have indicated that Peli1 negatively regulates mTOR signaling by mediating polyubiquitination of TSC1 and enhancing the stability of the TSC1/TSC2 complex in T cells [26]. Yet, in our mouse model, levels of TSC1 and TSC2 in kidney tissues did not significantly differ (Supplementary Fig. 6). Notably, kidneys from rtTA-Peli1 mice treated with doxycycline for 12 weeks displayed increased levels of phospho-S6 (Ser240/244) (p-S6), reflecting enhanced mTOR activation (Fig. 5a). Detection of a slight upregulation in S6K1, the kinase responsible for S6 protein phosphorylation, led us to investigate the effect of Peli1 on S6K1 activity. We further assessed p-S6 expression in kidney tissues from rtTA and rtTA-Peli1 mice after 24 weeks of doxycycline treatment using immunohistochemistry. A pronounced increase in p-S6 immunoreactivity intensity was observed in the kidney, especially within cyst epithelial regions of rtTA-Peli1 mice when compared to rtTA controls (Fig. 5b). In parallel, silencing Peli1 in the HK-2 human kidney proximal tubular cell line resulted in a notable decrease in both the abundance and the stability of endogenous S6K1 (Fig. 5c). We subsequently examined the interaction between Peli1 and S6K1 to elucidate how Peli1 modulates S6K1 regulation. Pull-down and co-immunoprecipitation experiments using co-expressed Peli1 and S6K1 in HK-2 cells revealed a direct association between these two proteins (Fig. 5d, e).

### Peli1 facilitates the stabilization of S6K1 via K63-linked ubiquitination

**Fig. 6:**
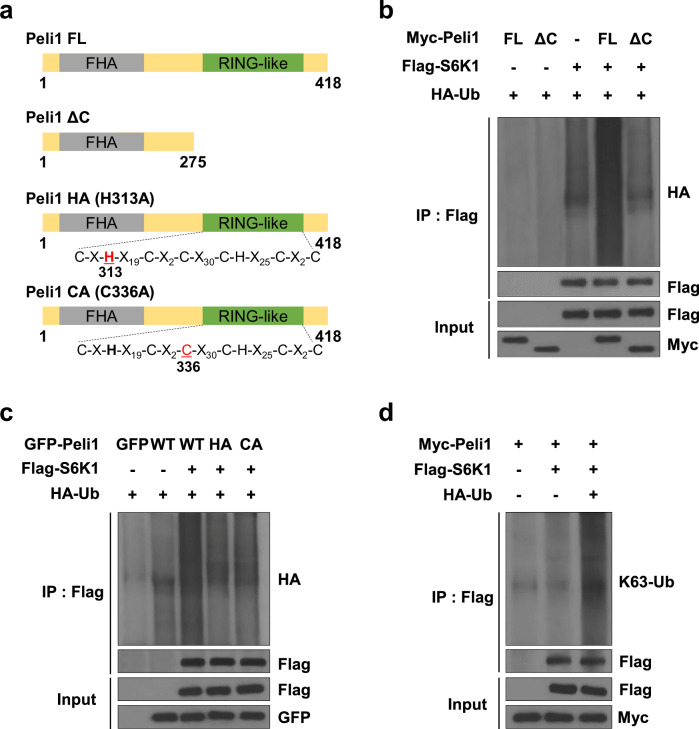
Peli1 stabilizes S6K1 by promoting lysine 63-linked ubiquitination. **a** Schematic diagrams illustrate wild-type and mutant Peli1 vectors. **b** Flag-S6K1 was transfected into 293T cells with either Myc-tagged WT or ΔC Peli1, or without Peli1, for 20 h before cell lysates were immunoprecipitated with an anti-Flag antibody. **c** 293T cells were transfected with Flag-S6K1 and/or HA-Ub in the presence or absence of GFP-tagged WT, HA (H313A), or CA (C336A) Peli1 for 20 h, followed by immunoprecipitation with an anti-Flag antibody. **d** K63-linked ubiquitination of S6K1 was analyzed in 293T cells after transfection with Myc-Peli1, Flag-S6K1, or HA-Ub.

We further investigated whether the E3 ligase Peli1 can mediate polyubiquitination of S6K1. Peli1 possesses a forkhead-associated (FHA) domain that facilitates substrate binding, as well as a RING-like domain responsible for its E3 ubiquitin ligase function [50]. To clarify whether the RING-like domain of Peli1 is essential for S6K1 ubiquitination, we employed a deletion variant lacking the RING domain (ΔC) and two RING mutants [H313A (HA) and C336A (CA)] (Fig. 6a). Our results demonstrated that overexpression of Peli1 WT, but not the Peli1 mutants, strongly enhanced S6K1 ubiquitination (Fig. 6b). Additionally, we used GFP-tagged Peli1 WT and two RING mutants co-expressed with HA-Ub. Only Peli1 WT expression initiated S6K1 ubiquitination, whereas the RING mutants had no effect (Fig. 6c), indicating that S6K1 ubiquitination is dependent on the E3 ligase activity of Peli1. Notably, we found that Peli1 primarily promoted K63-linked ubiquitination of S6K1 (Fig. 6d). Together, these results establish that Peli1 physically associates with S6K1 and facilitates its stabilization via RING-like domain-mediated K63-linked ubiquitination.

## Discussion

ADPKD is mainly caused by pathogenic germline variants in PKD1 (~80%) or PKD2 (~15%), with additional somatic “second hits” frequently arising in individual cysts [3]. As cysts enlarge, they trigger mechanical and cellular stress, disrupting primary-cilium/Ca2^+^ homeostasis and leading to DAMP release and activation of TLR2/4-NF-κB signaling in the surrounding renal epithelium; persistent inflammation can ultimately result in fibrosis [38, 51–53]. Given that Peli1 acts as a key amplifier in TLR/NF-κB and PI3K/AKT signaling pathways, we proposed that Peli1 serves as an upstream regulator connecting inflammatory stimuli with proliferative responses in cystic epithelial cells. Our findings demonstrate that Peli1 is a pivotal driver in polycystic kidney disease, promoting tubular epithelial proliferation and migration by facilitating K63-linked stabilization of S6K1 and activation of mTOR, thereby accelerating cyst progression (Graphical abstract). We also identified a correlation between elevated Peli1 expression and PKD progression, as well as poorer prognosis in ccRCC, supporting a mechanistic role for Peli1 in linking inflammatory signaling with mTOR-mediated proliferation in ADPKD and implicating it as a potential biomarker and therapeutic target at the ADPKD-RCC interface.

Abnormal regulation of TLRs has been shown to heighten the organism’s response to external stimuli, and such dysregulation has been reported in various human diseases, including cancer [54, 55]. Several studies have reported increased expression of TLRs and impaired TLR signaling pathways in RCC [56–59]. Furthermore, in patients with ADPKD, the rapid progression group exhibited higher TLR2 and TLR4 expression levels compared to those with slower disease progression [21, 38]. Peli1 is a key regulator of inflammatory responses modulating several signaling pathways upon stimulation by factors such as TLRs, NF-κB, and PI3K/AKT [23]. Specifically, Peli1 functions as a positive regulator of the TLR2- and TLR4-mediated signaling pathways within the MyD88-IRAK1-TAK1-TRAF6 and TRIF-TBK1 axes [50, 60]. Consistent with these findings, we observed upregulation of TLRs and Peli1 in kidney epithelial cell lines and public database analyses of ADPKD. In cystic kidney tissue from rtTA-Peli1 mice, NF-κB–driven cytokines, including IL-6, IL-1β, and CCL2, were found to be upregulated. Overall, our results further support that inflammation is a hallmark of ADPKD and designate Peli1 as a TLR-inducible mediator that enhances NF-κB-dependent cytokine expression and integrates innate immune signals.

Overexpression of Peli1 results in the development of psoriasis [24]. Psoriasis is a chronic inflammatory skin disorder that can lead to multiple complications. Common comorbidities include kidney disease, obesity, hypertension, and diabetes, which are recognized as cardiovascular (CV) risk factors [61]. Furthermore, the development of kidney disease is closely associated with risk factors commonly observed in individuals with psoriasis. Our findings show that elevated expression of Peli1 through pathogenic receptor activation promotes hyperplasia of tubular epithelial cells, a process likely contributing to PKD. We also identified BMD loss as a comorbidity associated with PKD. Although the period of observation was too short to adequately assess the development of psoriasis and cancer due to several complications, including BMD loss, Peli1 overexpression in a PKD mouse model may contribute to various comorbidities such as chronic inflammation and cancer.

Mechanistically, Peli1 overexpression initiates mTOR signaling through K63-linked ubiquitination of S6K1, thereby promoting cyst expansion and facilitating both the proliferation and migration of cyst epithelial cells. This observation aligns with the established function of the PI3K/AKT/mTOR pathway in driving both renal cyst development and tumorigenesis [8, 32]. Notably, while earlier studies in T cells have shown that Peli1 can suppress mTOR activity by polyubiquitinating TSC1 and stabilizing the TSC1/TSC2 complex, our findings revealed no alterations in TSC1/TSC2 expression, indicating that Peli1 may have cell type-specific actions depending on its substrate proteins [24–26, 34, 62, 63]. In both murine PKD models and human ADPKD tissues, aberrant mTOR activation was apparent, as evidenced by enhanced phosphorylation of p70S6K, S6, 4E-BP1, and AKT in cystic epithelia as well as adjacent non-cystic cells [10, 64]. Both preclinical and clinical research efforts have targeted mTOR signaling in ADPKD, expanding to modulation of upstream effectors like AMPK and glycosphingolipid biosynthesis [65, 66]. Agents such as rapamycin and its analogs have demonstrated therapeutic benefit in preclinical PKD and ccRCC studies. Nonetheless, their clinical utility is hampered by long-term adverse effects and nephrotoxicity [67–69]. These obstacles highlight the necessity for further investigation into novel single agents or combinatorial regimens that target alternative molecular pathways, aiming to improve treatment outcomes in ADPKD and RCC. To date, there are no pharmacologic agents that directly target Peli1. Nevertheless, recent progress in drugging E3 ubiquitin ligases, including advances in molecular glues, peptide-based inhibitors, and proteolysis-targeting chimeras (PROTACs) [23], suggests promising strategies for the selective modulation of Peli1 activity. These approaches may allow for indirect targeting of Peli1 or modulation of its downstream signaling.

Our study identified the Peli1-S6K1-mTOR axis as a pivotal inflammatory pathway driving cytogenesis in ADPKD, demonstrated using both rtTA-Peli1 mouse models and human ccRCC patient samples. Nevertheless, several limitations must be considered. First, the absence of tubule-specific loss-of-function and genetic epistasis experiments, such as S6K1 perturbation, restricts the in vivo validation of this axis. Second, the use of a single-center ccRCC cohort selected by tissue availability results in insufficient human validation. Recent single-cell multiomic analyses of human ADPKD have revealed considerable cellular heterogeneity [70]. Additional mechanistic investigations are necessary to clarify how Peli1 functions in various renal cell types and to determine the specific factors that activate Peli1 through TLR signaling.

In conclusion, our study demonstrates that Peli1 is a TLR-responsive E3 ubiquitin ligase that promotes the progression of ADPKD. Consistent with PKD1-deficient mouse models, Peli1-overexpressing mice showed increased proliferation of renal TECs, resulting in cyst development. Mechanistically, Peli1 induces K63-linked ubiquitination of S6K1, which activates the mTOR pathway and emphasizes the key regulatory role of ubiquitination in kidney disease. Together, our results identify Peli1 as a mechanistic link between inflammatory progression in ADPKD and its malignant transformation, supporting Peli1 as a promising therapeutic target for ADPKD and potentially RCC.

## Supplementary information

Supplementary Figures and Figure Legends

Full unedited gels for figures

## Data Availability

All data supporting our study are available in the main manuscript or the supplementary materials. We will also share models, methods, protocols, and other relevant materials and resources related to the article, to the extent possible.
