# Supplementary material for: Pellino1-mTOR/S6K1 signaling axis is a key pathogenesis for the development of polycystic kidney disease
Source: Cell Death Dis. 2026 Mar 5;17(1):296. doi: 10.1038/s41419-026-08479-6 (PMC13040062; doi:10.1038/s41419-026-08479-6)
Supplement: Supplementary file 1 — Supplementary Figures and Figure Legends [file 41419_2026_8479_MOESM1_ESM.pptx]

## Slide 1
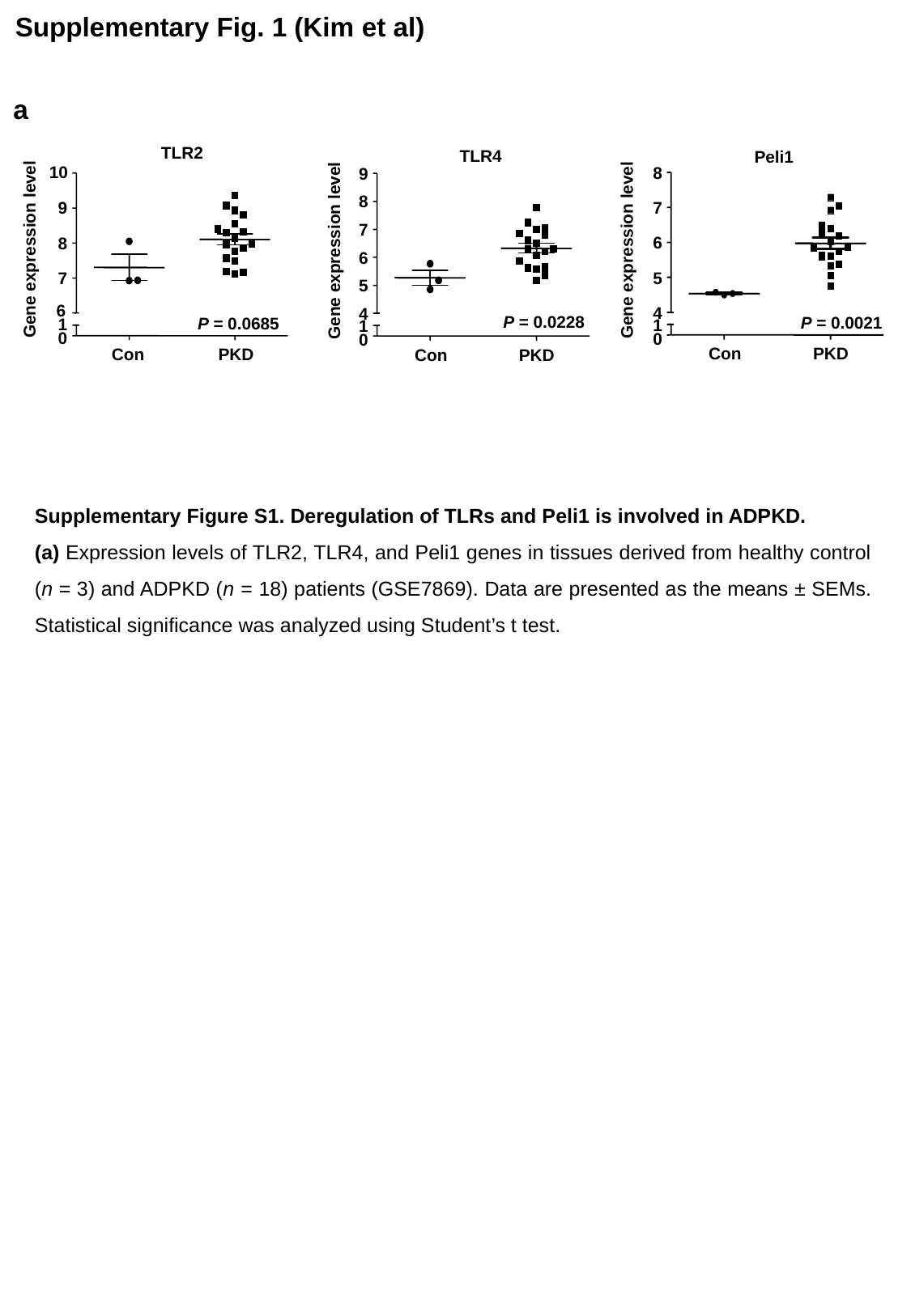

Supplementary Fig. 1 (Kim et al)
a
TLR2
10
Gene expression level
P = 0.0685
9
8
7
1
0
Con
PKD
TLR4
9
Gene expression level
8
7
6
5
6
4
P = 0.0228
1
0
Con
PKD
Peli1
8
Gene expression level
7
6
5
4
P = 0.0021
1
0
Con
PKD
Supplementary Figure S1. Deregulation of TLRs and Peli1 is involved in ADPKD.
(a) Expression levels of TLR2, TLR4, and Peli1 genes in tissues derived from healthy control (n = 3) and ADPKD (n = 18) patients (GSE7869). Data are presented as the means ± SEMs. Statistical significance was analyzed using Student’s t test.

## Slide 2
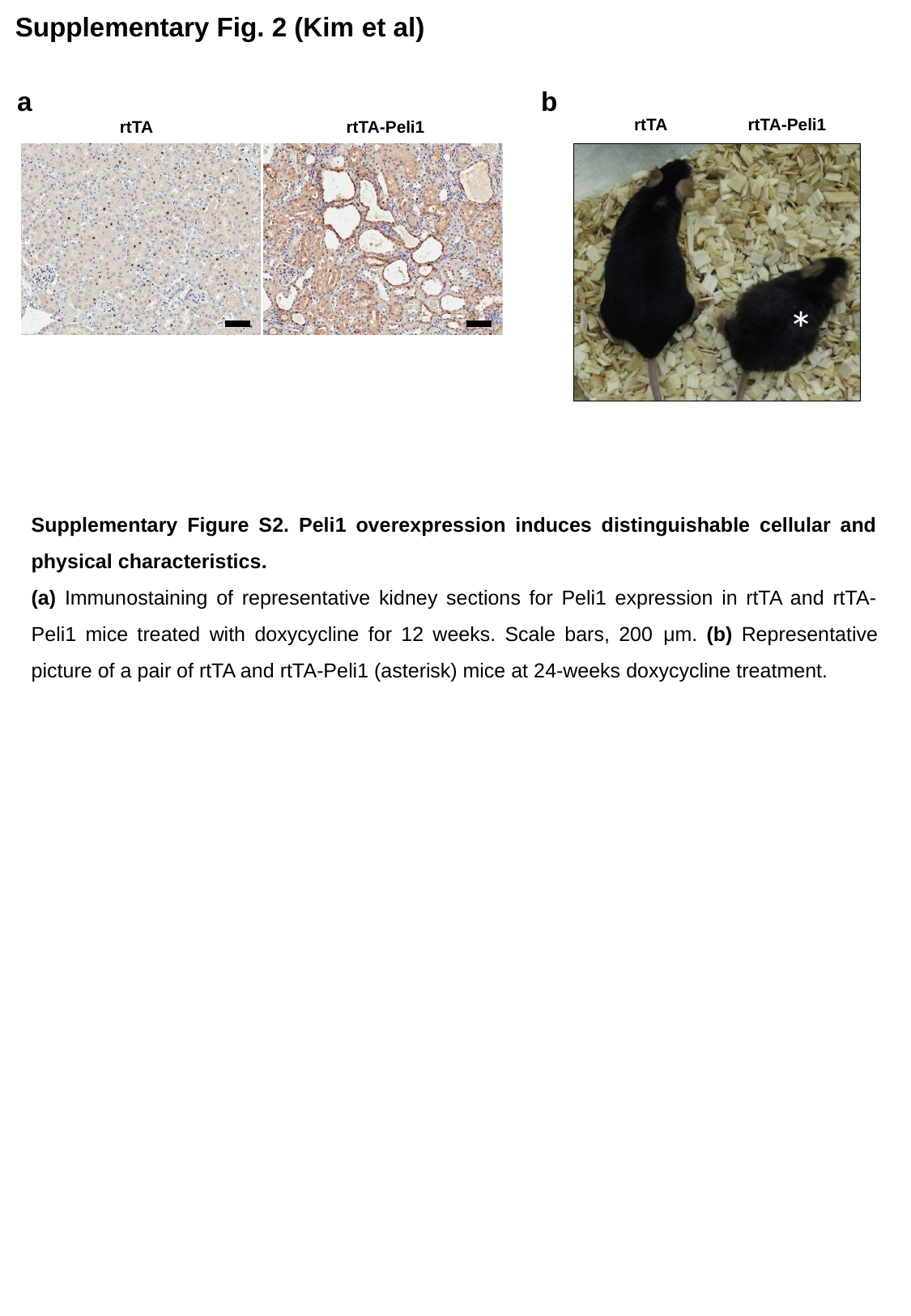

Supplementary Fig. 2 (Kim et al)
a
b
rtTA
rtTA-Peli1
rtTA
rtTA-Peli1
*
Supplementary Figure S2. Peli1 overexpression induces distinguishable cellular and physical characteristics.
(a) Immunostaining of representative kidney sections for Peli1 expression in rtTA and rtTA-Peli1 mice treated with doxycycline for 12 weeks. Scale bars, 200 μm. (b) Representative picture of a pair of rtTA and rtTA-Peli1 (asterisk) mice at 24-weeks doxycycline treatment.

## Slide 3
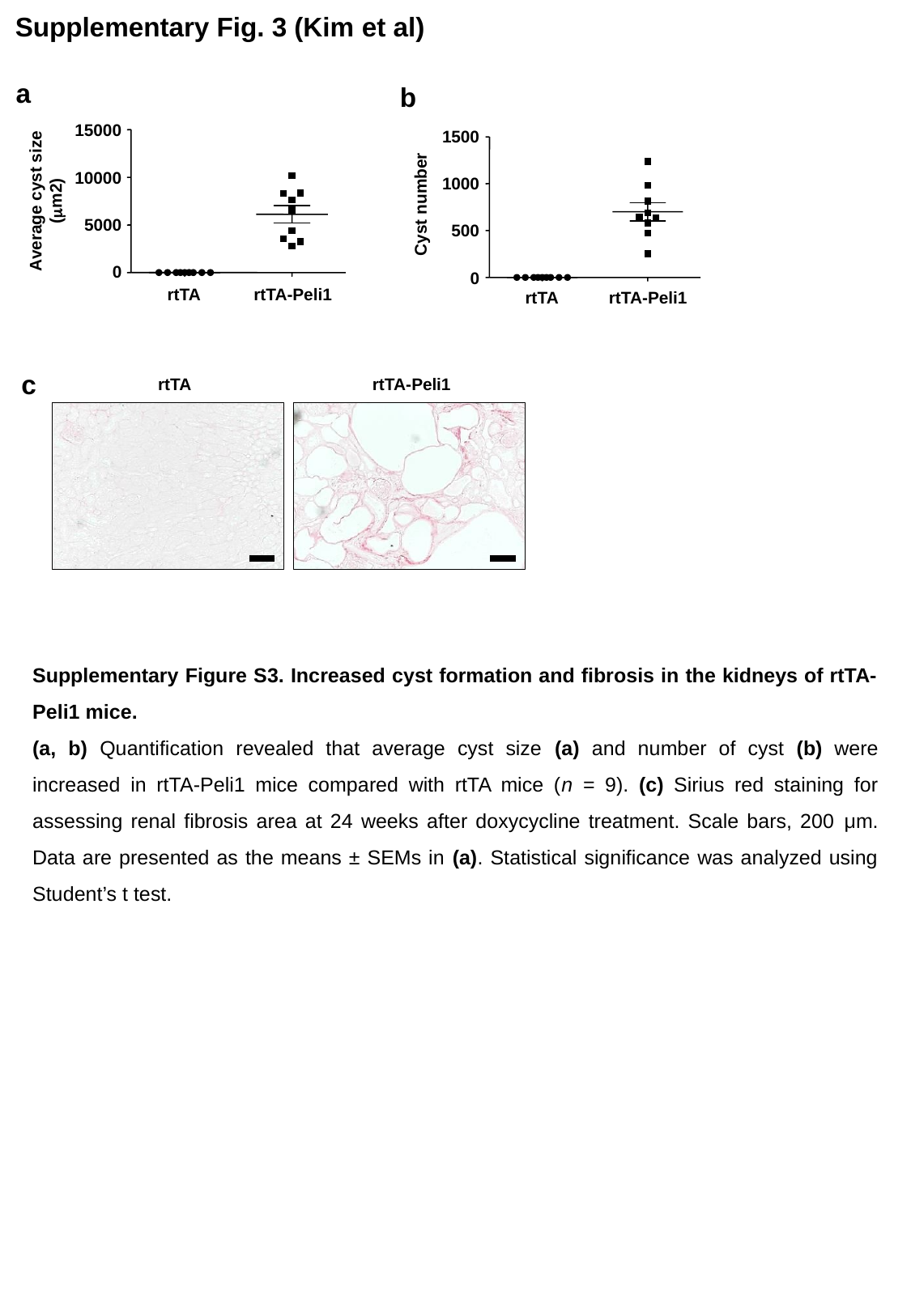

Supplementary Fig. 3 (Kim et al)
a
b
15000
10000
5000
0
rtTA
rtTA-Peli1
Average cyst size (mm2)
1500
1000
Cyst number
500
0
rtTA
rtTA-Peli1
c
rtTA
rtTA-Peli1
Supplementary Figure S3. Increased cyst formation and fibrosis in the kidneys of rtTA-Peli1 mice.
(a, b) Quantification revealed that average cyst size (a) and number of cyst (b) were increased in rtTA-Peli1 mice compared with rtTA mice (n = 9). (c) Sirius red staining for assessing renal fibrosis area at 24 weeks after doxycycline treatment. Scale bars, 200 μm. Data are presented as the means ± SEMs in (a). Statistical significance was analyzed using Student’s t test.

## Slide 4
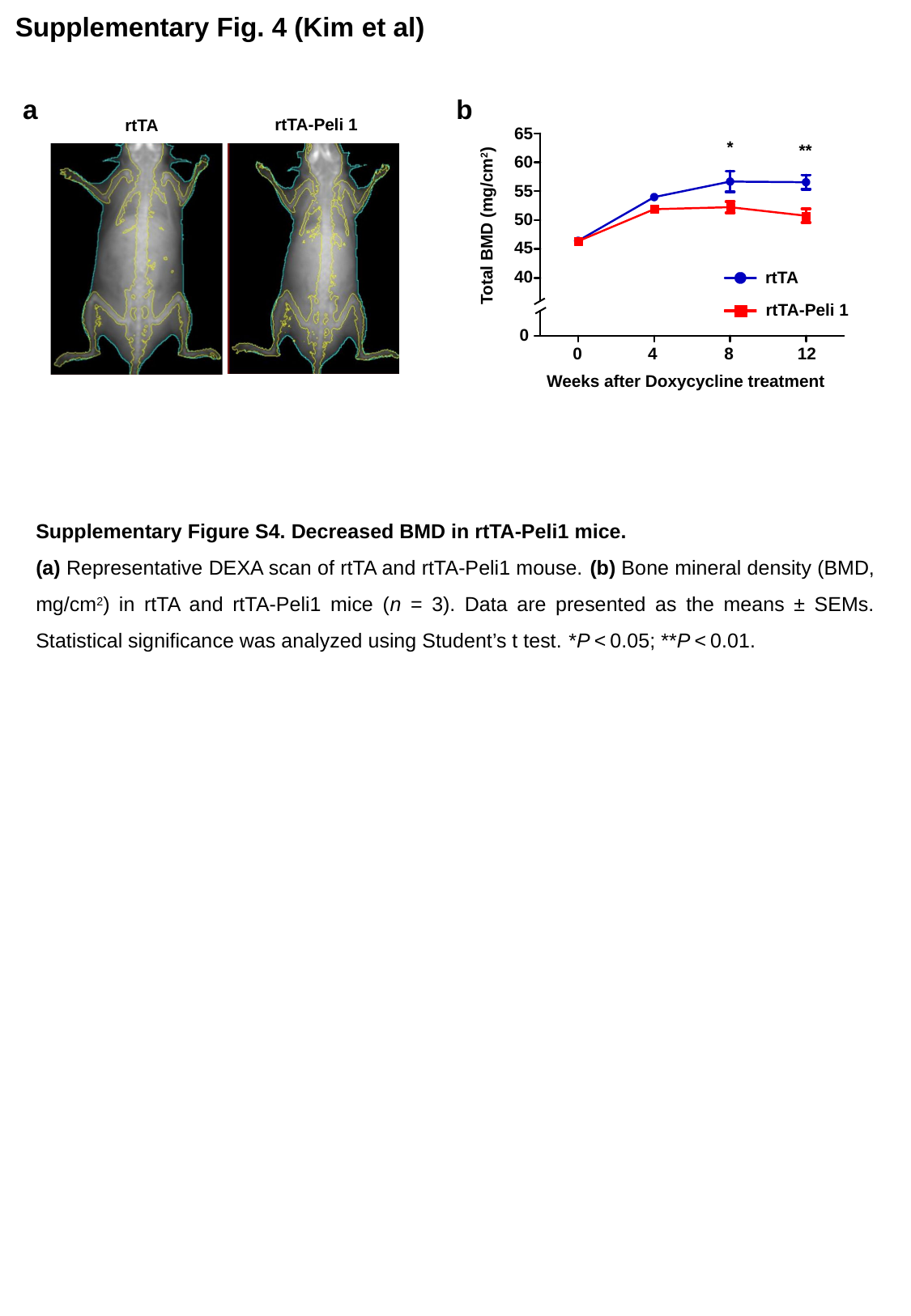

Supplementary Fig. 4 (Kim et al)
a
b
rtTA-Peli 1
rtTA
65
Total BMD (mg/cm2)
60
55
50
45
40
0
0
4
8
12
*
**
rtTA
rtTA-Peli 1
Weeks after Doxycycline treatment
Supplementary Figure S4. Decreased BMD in rtTA-Peli1 mice.
(a) Representative DEXA scan of rtTA and rtTA-Peli1 mouse. (b) Bone mineral density (BMD, mg/cm2) in rtTA and rtTA-Peli1 mice (n = 3). Data are presented as the means ± SEMs. Statistical significance was analyzed using Student’s t test. *P < 0.05; **P < 0.01.

## Slide 5
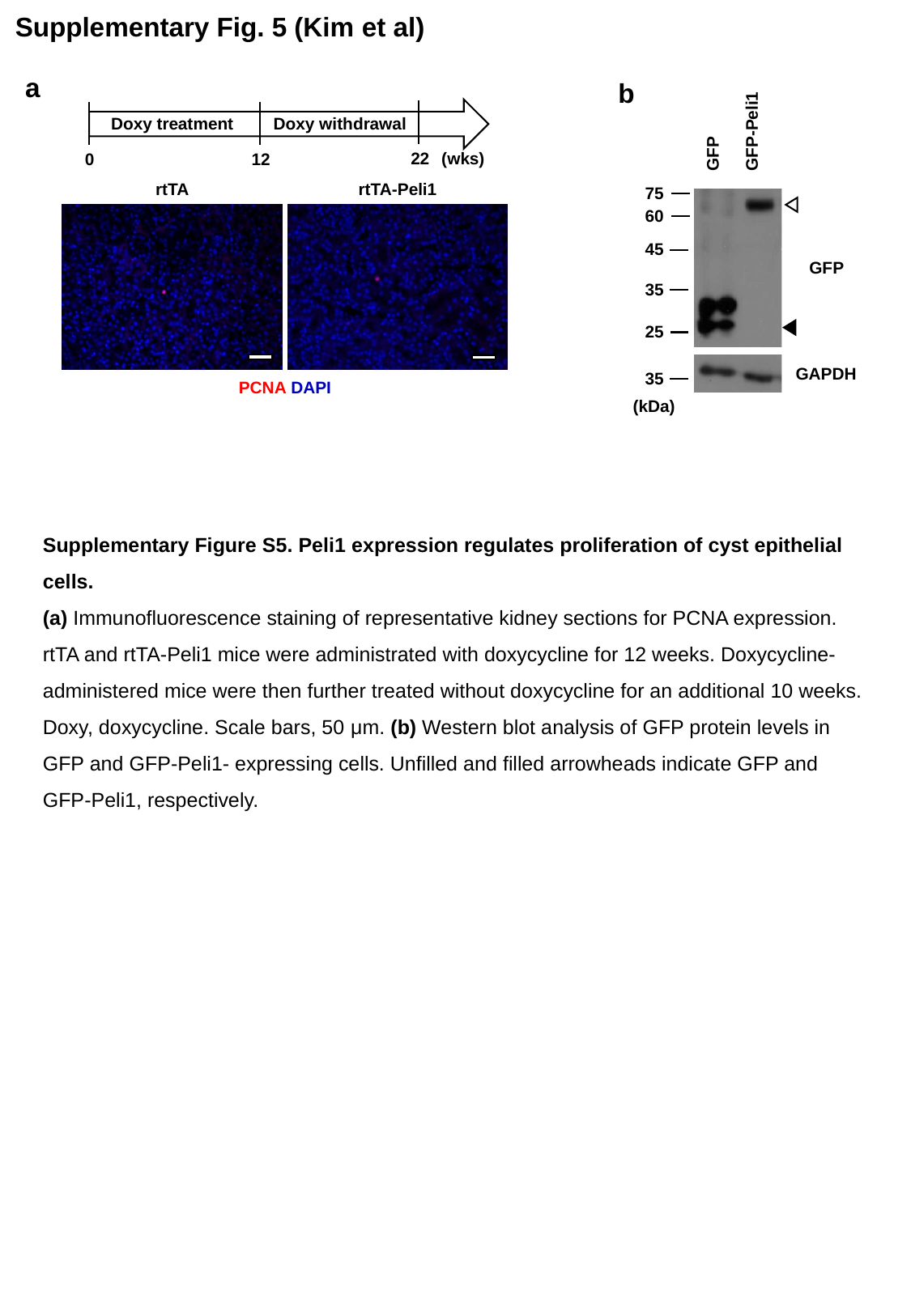

Supplementary Fig. 5 (Kim et al)
a
b
GFP-Peli1
GFP
75
60
45
GFP
35
25
GAPDH
35
(kDa)
Doxy withdrawal
Doxy treatment
22
(wks)
0
12
rtTA
rtTA-Peli1
PCNA DAPI
Supplementary Figure S5. Peli1 expression regulates proliferation of cyst epithelial cells.
(a) Immunofluorescence staining of representative kidney sections for PCNA expression. rtTA and rtTA-Peli1 mice were administrated with doxycycline for 12 weeks. Doxycycline-administered mice were then further treated without doxycycline for an additional 10 weeks. Doxy, doxycycline. Scale bars, 50 μm. (b) Western blot analysis of GFP protein levels in GFP and GFP-Peli1- expressing cells. Unfilled and filled arrowheads indicate GFP and GFP-Peli1, respectively.

## Slide 6
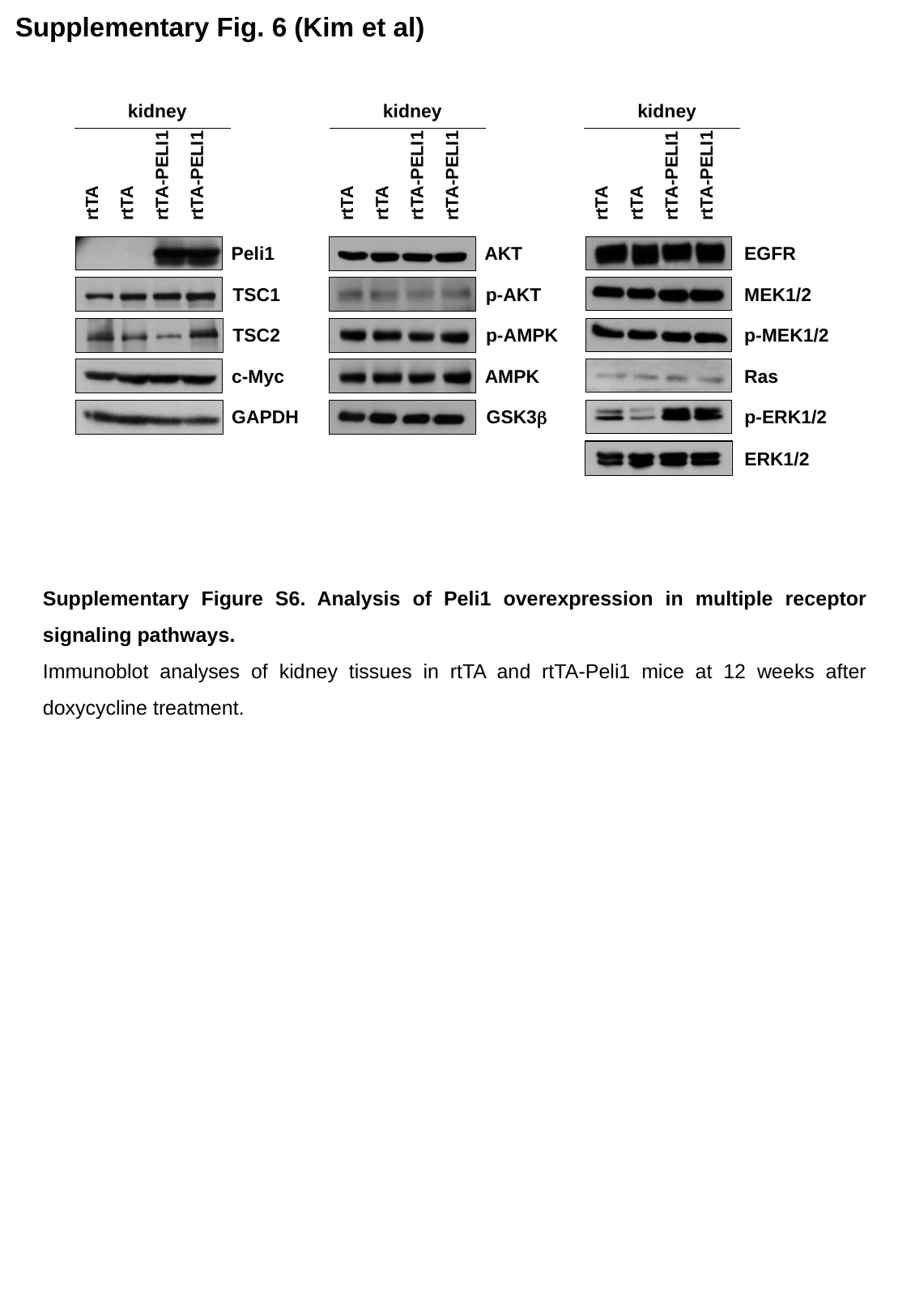

Supplementary Fig. 6 (Kim et al)
kidney
kidney
kidney
rtTA-PELI1
rtTA-PELI1
rtTA-PELI1
rtTA-PELI1
rtTA-PELI1
rtTA-PELI1
rtTA
rtTA
rtTA
rtTA
rtTA
rtTA
Peli1
AKT
EGFR
TSC1
p-AKT
MEK1/2
TSC2
p-AMPK
p-MEK1/2
c-Myc
AMPK
Ras
GAPDH
GSK3b
p-ERK1/2
ERK1/2
Supplementary Figure S6. Analysis of Peli1 overexpression in multiple receptor signaling pathways.
Immunoblot analyses of kidney tissues in rtTA and rtTA-Peli1 mice at 12 weeks after doxycycline treatment.

## Slide 7
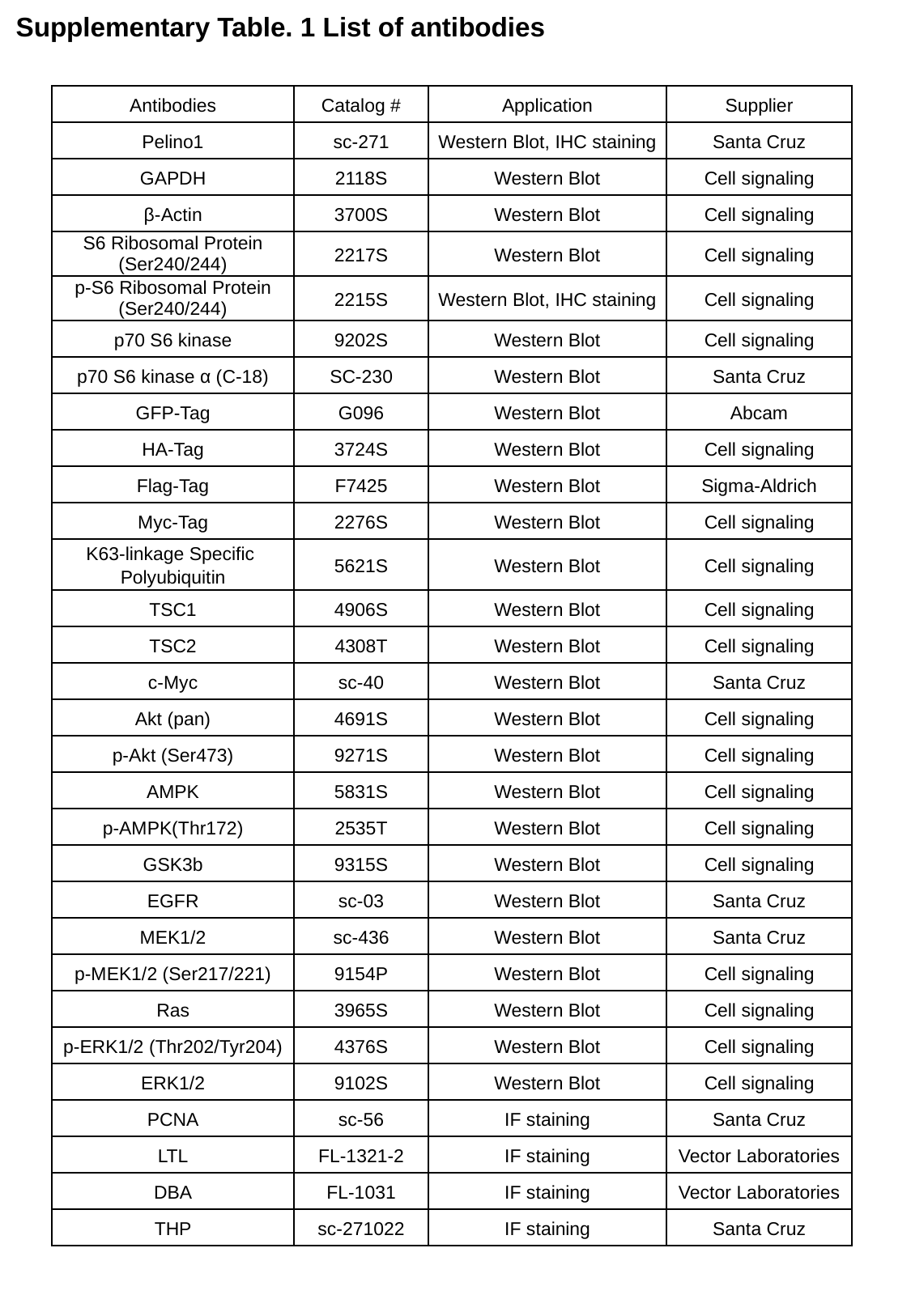

Supplementary Table. 1 List of antibodies
| Antibodies | Catalog # | Application | Supplier |
| --- | --- | --- | --- |
| Pelino1 | sc-271 | Western Blot, IHC staining | Santa Cruz |
| GAPDH | 2118S | Western Blot | Cell signaling |
| β-Actin | 3700S | Western Blot | Cell signaling |
| S6 Ribosomal Protein (Ser240/244) | 2217S | Western Blot | Cell signaling |
| p-S6 Ribosomal Protein (Ser240/244) | 2215S | Western Blot, IHC staining | Cell signaling |
| p70 S6 kinase | 9202S | Western Blot | Cell signaling |
| p70 S6 kinase α (C-18) | SC-230 | Western Blot | Santa Cruz |
| GFP-Tag | G096 | Western Blot | Abcam |
| HA-Tag | 3724S | Western Blot | Cell signaling |
| Flag-Tag | F7425 | Western Blot | Sigma-Aldrich |
| Myc-Tag | 2276S | Western Blot | Cell signaling |
| K63-linkage Specific Polyubiquitin | 5621S | Western Blot | Cell signaling |
| TSC1 | 4906S | Western Blot | Cell signaling |
| TSC2 | 4308T | Western Blot | Cell signaling |
| c-Myc | sc-40 | Western Blot | Santa Cruz |
| Akt (pan) | 4691S | Western Blot | Cell signaling |
| p-Akt (Ser473) | 9271S | Western Blot | Cell signaling |
| AMPK | 5831S | Western Blot | Cell signaling |
| p-AMPK(Thr172) | 2535T | Western Blot | Cell signaling |
| GSK3b | 9315S | Western Blot | Cell signaling |
| EGFR | sc-03 | Western Blot | Santa Cruz |
| MEK1/2 | sc-436 | Western Blot | Santa Cruz |
| p-MEK1/2 (Ser217/221) | 9154P | Western Blot | Cell signaling |
| Ras | 3965S | Western Blot | Cell signaling |
| p-ERK1/2 (Thr202/Tyr204) | 4376S | Western Blot | Cell signaling |
| ERK1/2 | 9102S | Western Blot | Cell signaling |
| PCNA | sc-56 | IF staining | Santa Cruz |
| LTL | FL-1321-2 | IF staining | Vector Laboratories |
| DBA | FL-1031 | IF staining | Vector Laboratories |
| THP | sc-271022 | IF staining | Santa Cruz |

## Slide 8
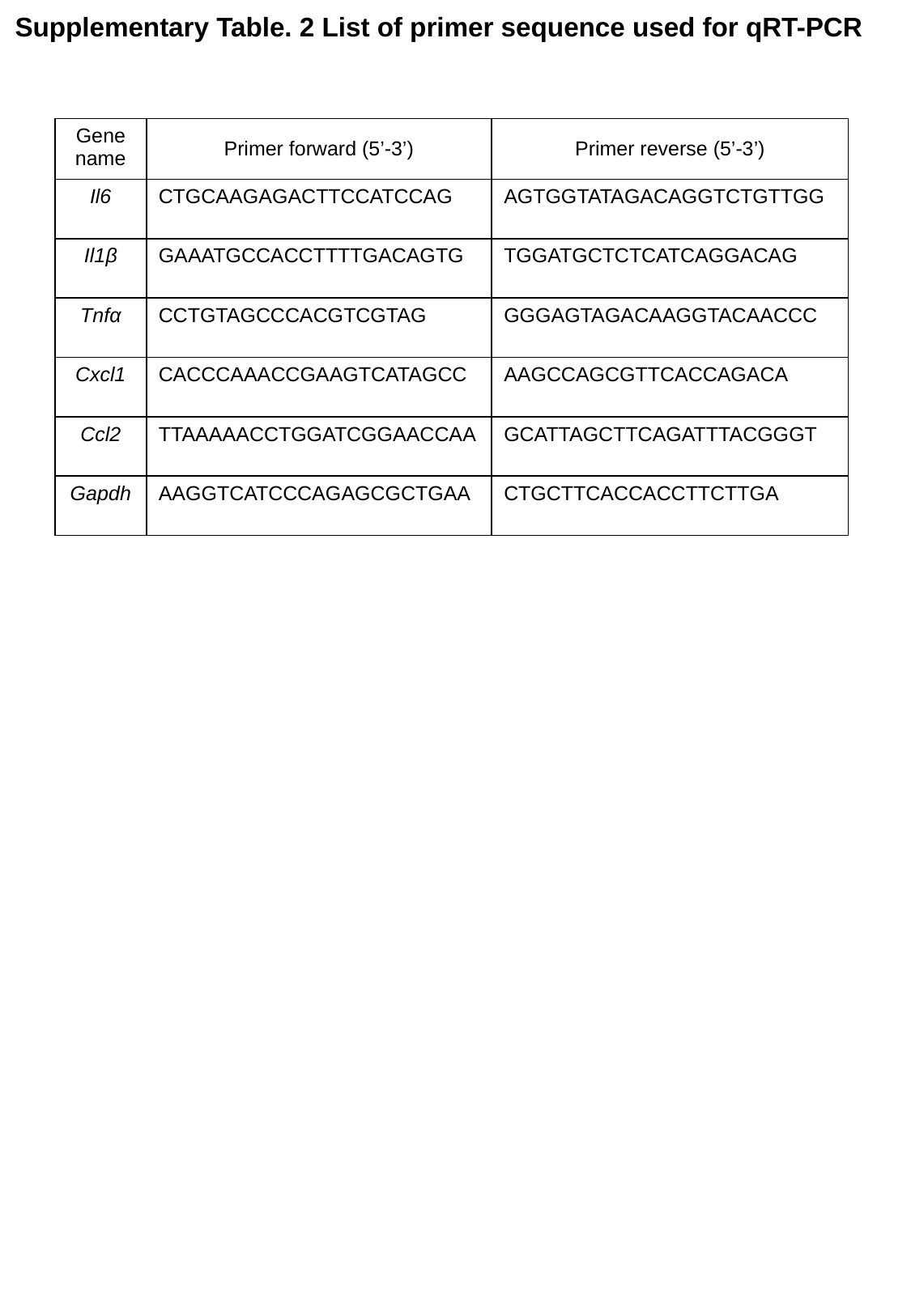

Supplementary Table. 2 List of primer sequence used for qRT-PCR
| Gene name | Primer forward (5’-3’) | Primer reverse (5’-3’) |
| --- | --- | --- |
| Il6 | CTGCAAGAGACTTCCATCCAG | AGTGGTATAGACAGGTCTGTTGG |
| Il1β | GAAATGCCACCTTTTGACAGTG | TGGATGCTCTCATCAGGACAG |
| Tnfα | CCTGTAGCCCACGTCGTAG | GGGAGTAGACAAGGTACAACCC |
| Cxcl1 | CACCCAAACCGAAGTCATAGCC | AAGCCAGCGTTCACCAGACA |
| Ccl2 | TTAAAAACCTGGATCGGAACCAA | GCATTAGCTTCAGATTTACGGGT |
| Gapdh | AAGGTCATCCCAGAGCGCTGAA | CTGCTTCACCACCTTCTTGA |
