# Supplementary material for: Pellino1-mTOR/S6K1 signaling axis is a key pathogenesis for the development of polycystic kidney disease
Source: Cell Death Dis. 2026 Mar 5;17(1):296. doi: 10.1038/s41419-026-08479-6 (PMC13040062; doi:10.1038/s41419-026-08479-6)
Supplement: Supplementary file 2 — Full unedited gels for figures [file 41419_2026_8479_MOESM2_ESM.pptx]

## Slide 1
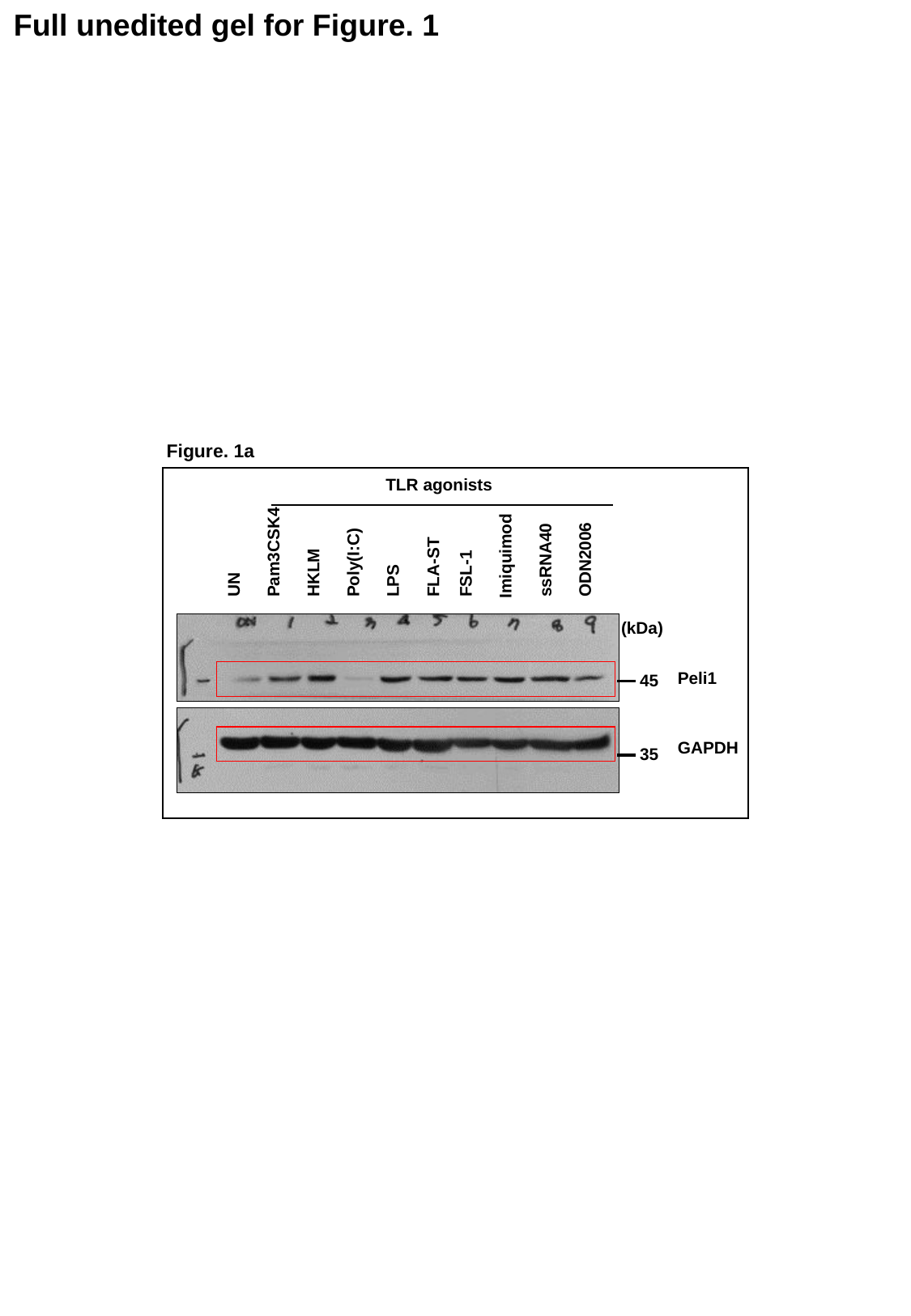

Full unedited gel for Figure. 1
Figure. 1a
TLR agonists
UN
Pam3CSK4
HKLM
Poly(I:C)
LPS
FLA-ST
FSL-1
ssRNA40
ODN2006
Imiquimod
(kDa)
Peli1
45
GAPDH
35

## Slide 2
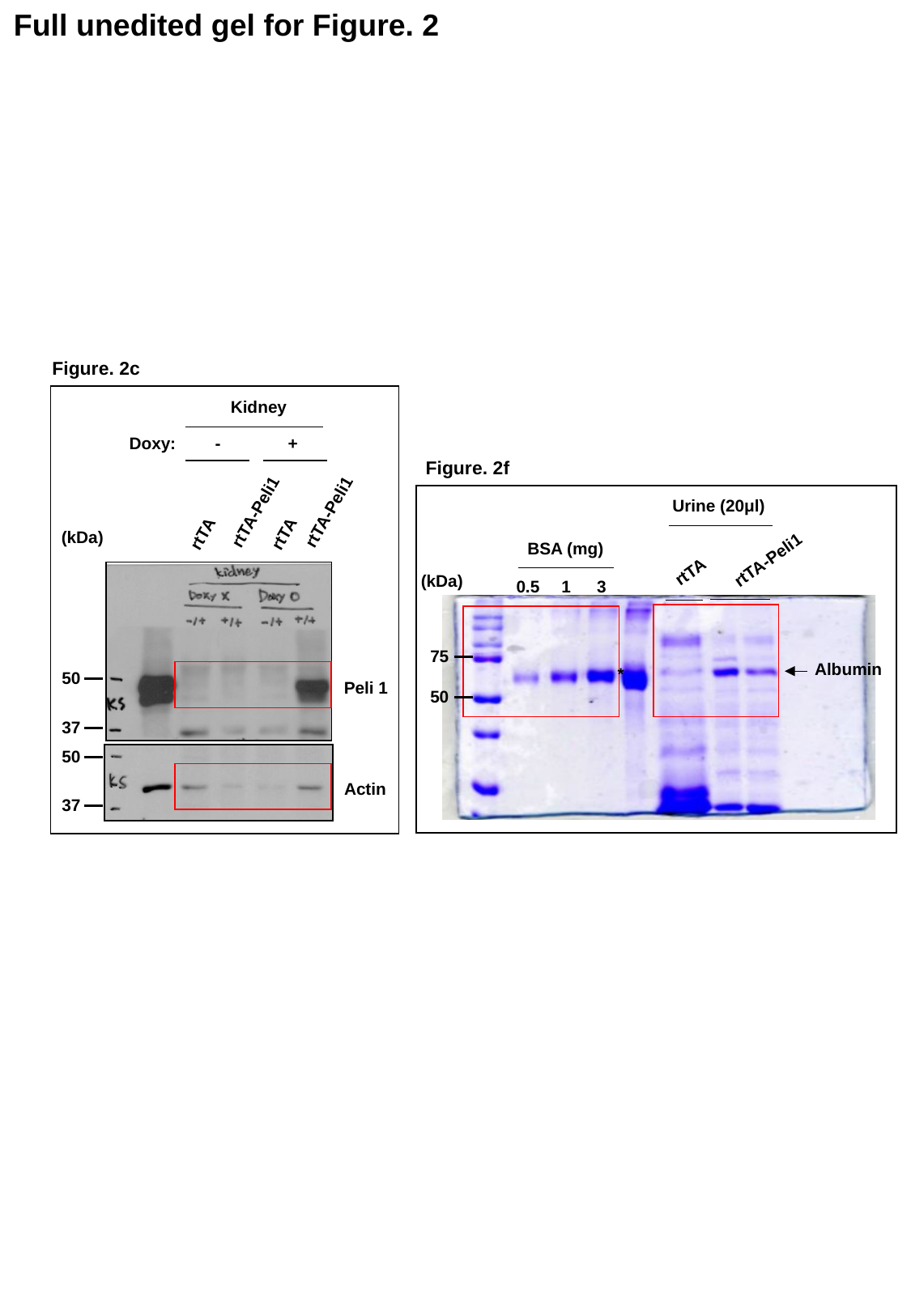

Full unedited gel for Figure. 2
Figure. 2c
Kidney
Doxy:
-
+
Figure. 2f
Urine (20μl)
rtTA-Peli1
rtTA-Peli1
rtTA
rtTA
(kDa)
BSA (mg)
rtTA-Peli1
rtTA
(kDa)
3
0.5
1
75
Albumin
*
50
Peli 1
50
37
50
Actin
37

## Slide 3
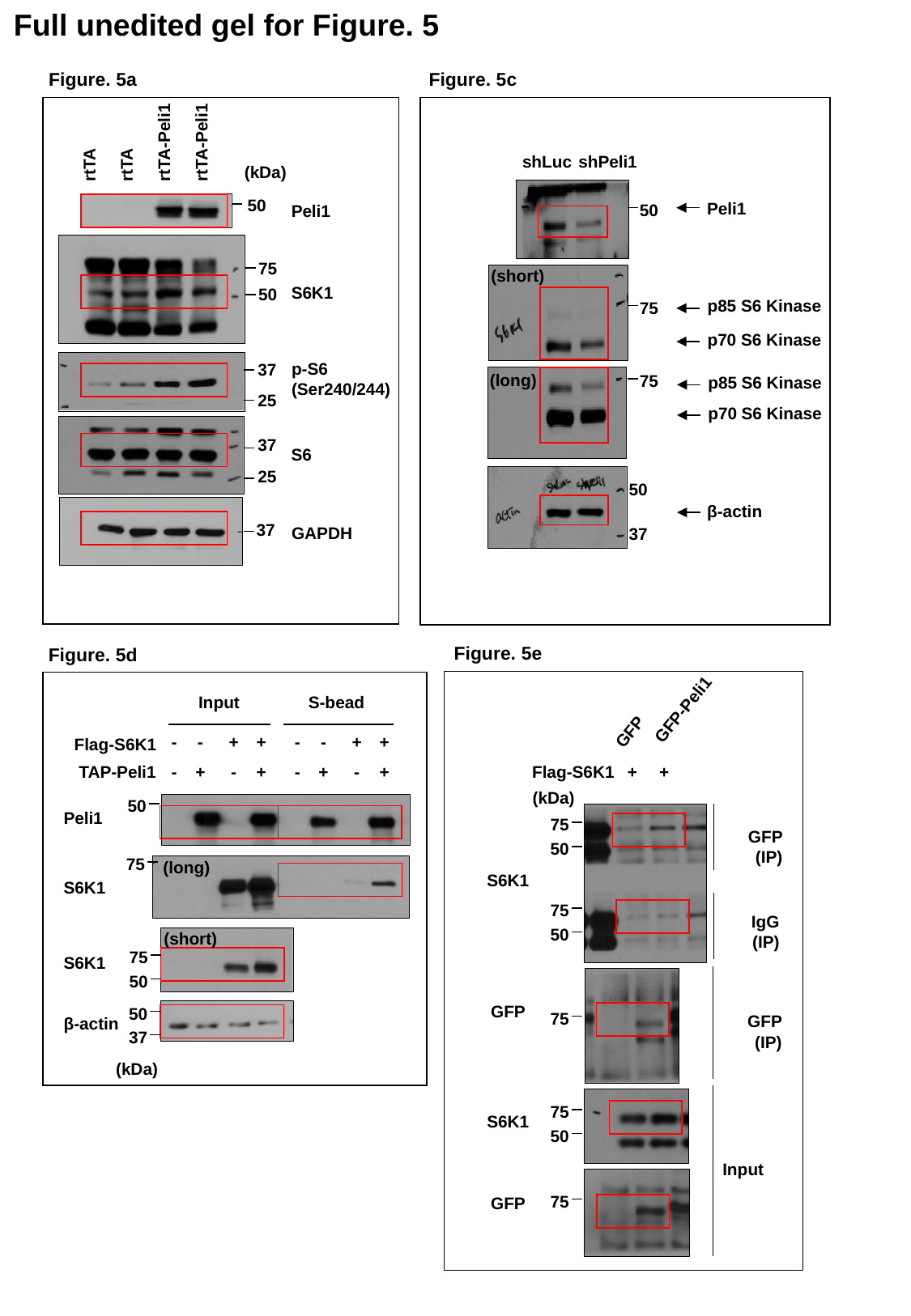

Full unedited gel for Figure. 5
Figure. 5a
Figure. 5c
rtTA-Peli1
rtTA-Peli1
shLuc
shPeli1
rtTA
rtTA
(kDa)
50
Peli1
50
Peli1
75
(short)
S6K1
50
p85 S6 Kinase
75
p70 S6 Kinase
p-S6
(Ser240/244)
37
(long)
75
p85 S6 Kinase
25
p70 S6 Kinase
37
S6
25
50
β-actin
37
GAPDH
37
Figure. 5e
Figure. 5d
GFP-Peli1
S-bead
Input
GFP
-
-
+
+
-
-
+
+
Flag-S6K1
-
+
-
+
-
+
-
+
TAP-Peli1
+
+
Flag-S6K1
(kDa)
50
Peli1
75
GFP (IP)
50
75
(long)
S6K1
S6K1
75
IgG (IP)
50
(short)
75
S6K1
50
GFP
50
75
GFP (IP)
β-actin
37
(kDa)
75
S6K1
50
Input
75
GFP

## Slide 4
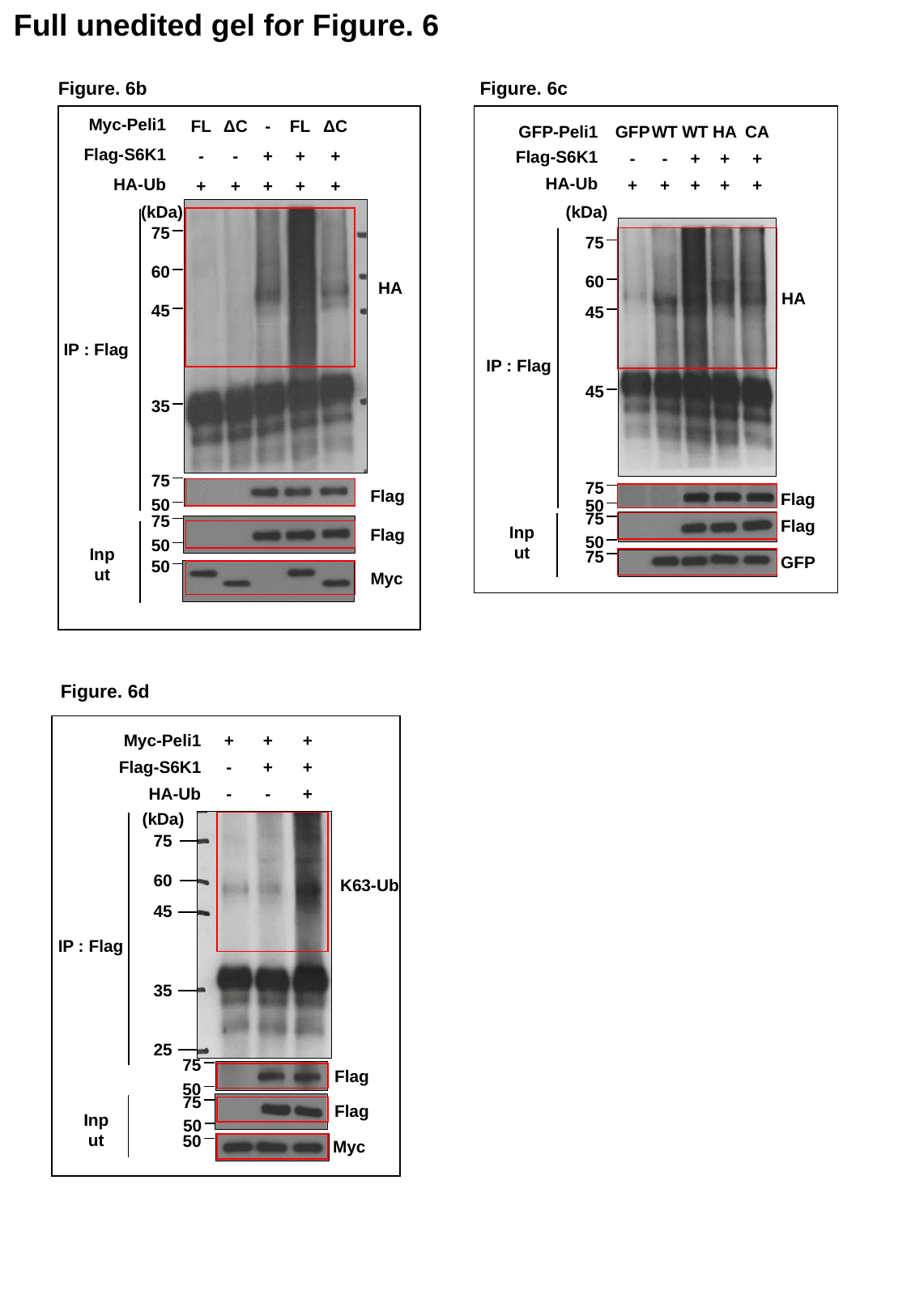

Full unedited gel for Figure. 6
Figure. 6b
Figure. 6c
Myc-Peli1
FL
-
+
ΔC
-
+
-
+
+
FL
+
+
ΔC
+
+
GFP-Peli1
GFP
WT
WT
HA
CA
Flag-S6K1
Flag-S6K1
-
-
+
+
+
HA-Ub
HA-Ub
+
+
+
+
+
(kDa)
(kDa)
75
75
60
60
HA
HA
45
45
IP : Flag
IP : Flag
45
35
75
75
Flag
Flag
50
50
75
75
Flag
Input
Flag
50
50
Input
75
GFP
50
Myc
Figure. 6d
Myc-Peli1
+
-
-
+
+
-
+
+
+
Flag-S6K1
HA-Ub
(kDa)
75
K63-Ub
60
45
IP : Flag
35
25
75
Flag
50
75
Flag
Input
50
50
Myc

## Slide 5
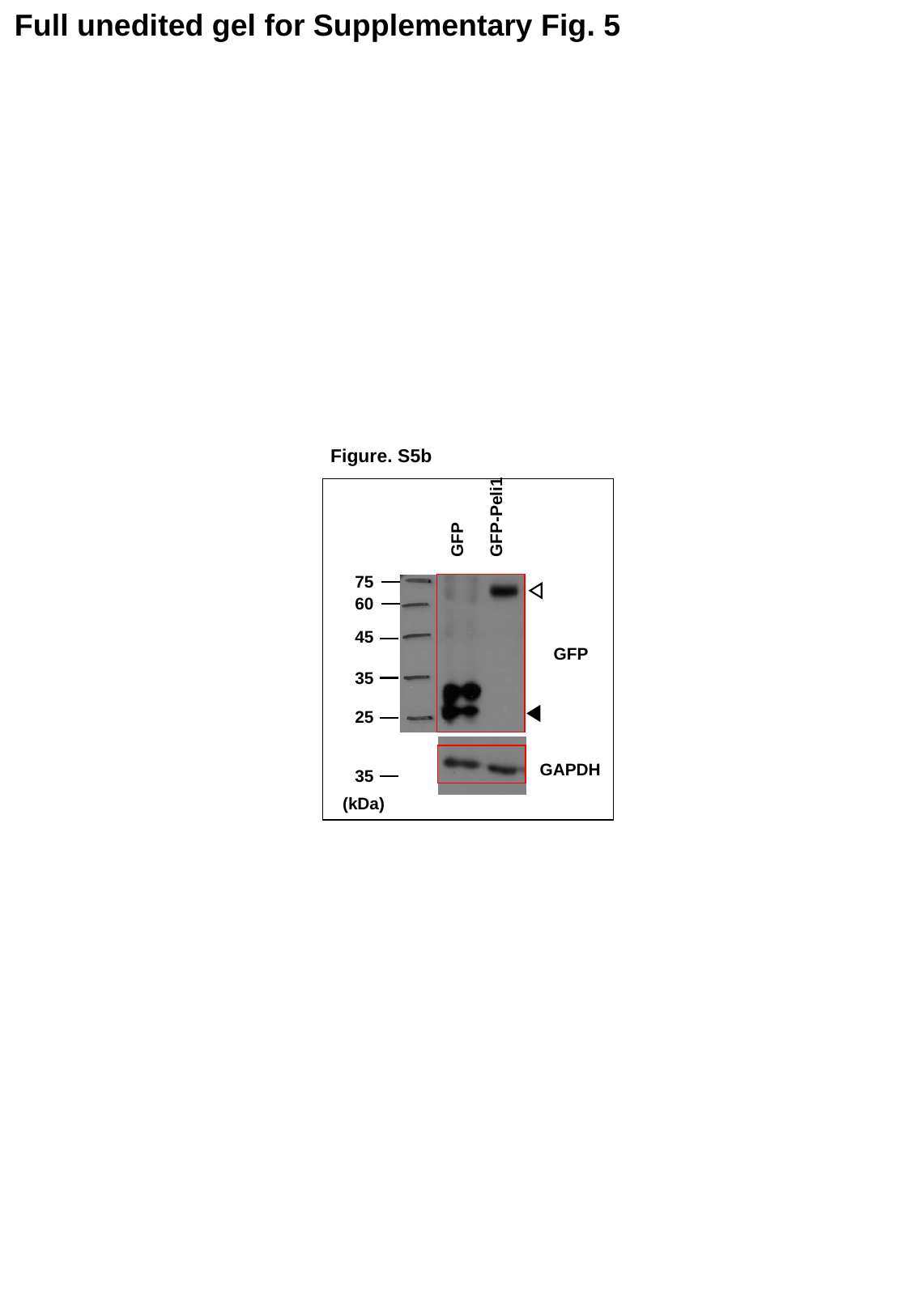

Full unedited gel for Supplementary Fig. 5
Figure. S5b
GFP-Peli1
GFP
75
60
45
GFP
35
25
GAPDH
35
(kDa)

## Slide 6
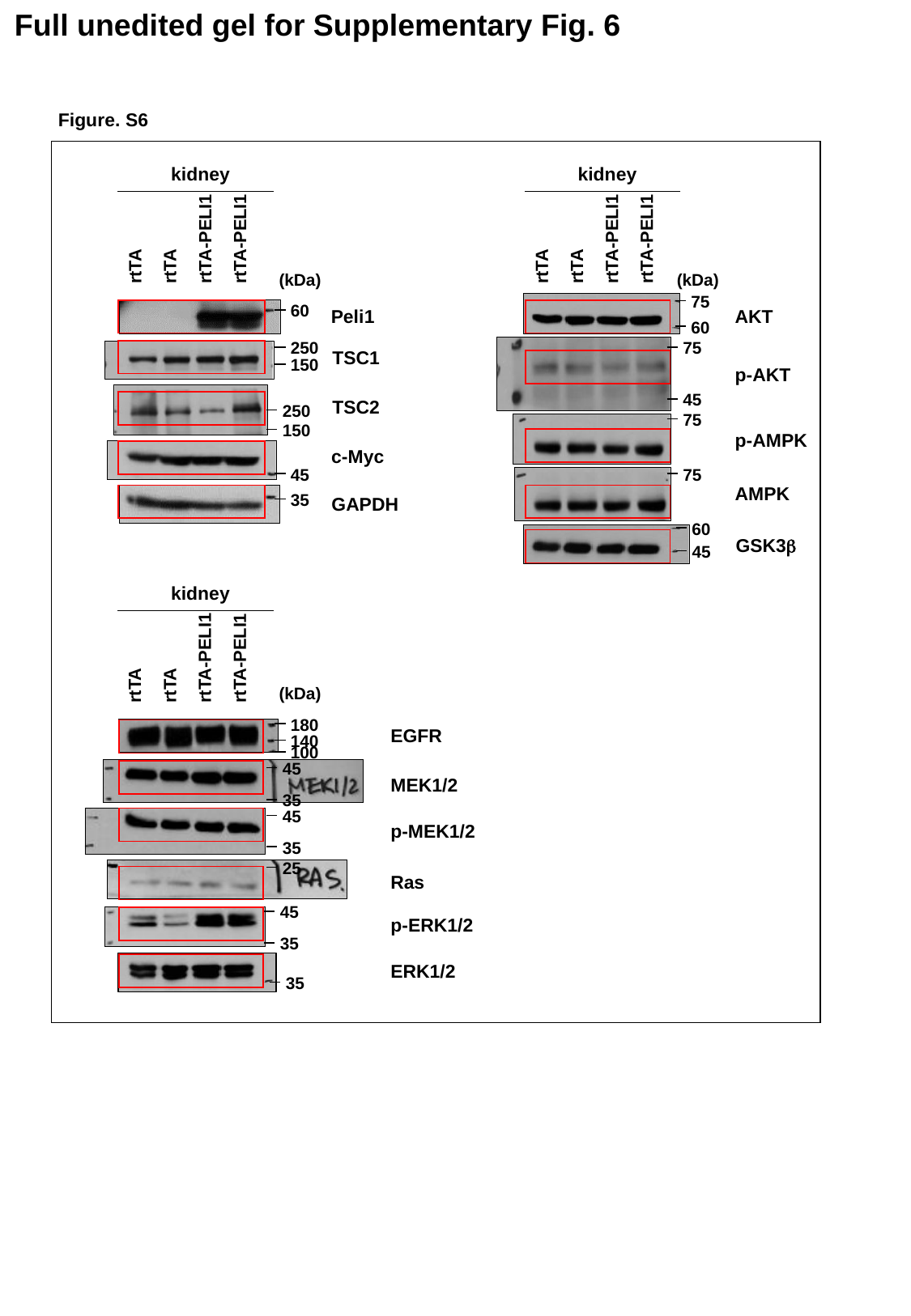

Full unedited gel for Supplementary Fig. 6
Figure. S6
kidney
rtTA-PELI1
rtTA-PELI1
rtTA
rtTA
(kDa)
60
Peli1
250
TSC1
150
TSC2
250
150
c-Myc
45
35
GAPDH
kidney
rtTA-PELI1
rtTA-PELI1
rtTA
rtTA
(kDa)
75
AKT
60
75
p-AKT
45
75
p-AMPK
75
AMPK
60
GSK3b
45
kidney
rtTA-PELI1
rtTA-PELI1
rtTA
rtTA
(kDa)
180
EGFR
140
100
45
MEK1/2
35
45
p-MEK1/2
35
25
Ras
45
p-ERK1/2
35
ERK1/2
35
